# Complete genome sequence and the expression pattern of plasmids of the model ethanologen *Zymomonas mobilis* ZM4 and its xylose-utilizing derivatives 8b and 2032

**DOI:** 10.1186/s13068-018-1116-x

**Published:** 2018-05-02

**Authors:** Shihui Yang, Jessica M. Vera, Jeff Grass, Giannis Savvakis, Oleg V. Moskvin, Yongfu Yang, Sean J. McIlwain, Yucai Lyu, Irene Zinonos, Alexander S. Hebert, Joshua J. Coon, Donna M. Bates, Trey K. Sato, Steven D. Brown, Michael E. Himmel, Min Zhang, Robert Landick, Katherine M. Pappas, Yaoping Zhang

**Affiliations:** 1 Hubei Collaborative Innovation Center for Green Transformation of Bio-resources, Environmental Microbial Technology Center of Hubei Province, Hubei Key Laboratory of Industrial Biotechnology, College of Life Sciences, Hubei University, Wuhan, 430062 China; 2 DOE-National Bioenergy Center, National Renewable Energy Laboratory (NREL), Golden, CO 80401 USA; 3 DOE-Great Lakes Bioenergy Research Center (GLBRC), University of Wisconsin-Madison, Madison, WI USA; 4 Department of Genetics & Biotechnology, Faculty of Biology, National and Kapodistrian University of Athens (NKUA), Panepistimiopolis, 15701 Athens, Greece; 5 DOE-BioEnergy Science Center, Oak Ridge National Laboratory (ORNL), Oak Ridge, TN 37831 USA; 6 DOE-Biosciences Division, Oak Ridge National Laboratory (ORNL), Oak Ridge, TN 37831 USA; 7 DOE-Biosciences Center, National Renewable Energy Laboratory (NREL), Golden, CO 80401 USA; 8 Present Address: China Three Gorges University, Yichang, 443002 Hubei China; 9 Present Address: LanzaTech, Inc., Skokie, IL 60077 USA

**Keywords:** *Zymomonas mobilis*, Plasmid, Genome, Genome resequencing, Annotation, RNA-Seq, Copy number, Hydrolysate, Fermentation

## Abstract

**Background:**

*Zymomonas mobilis* is a natural ethanologen being developed and deployed as an industrial biofuel producer. To date, eight *Z. mobilis* strains have been completely sequenced and found to contain 2–8 native plasmids. However, systematic verification of predicted *Z. mobilis* plasmid genes and their contribution to cell fitness has not been hitherto addressed. Moreover, the precise number and identities of plasmids in *Z. mobilis* model strain ZM4 have been unclear. The lack of functional information about plasmid genes in ZM4 impedes ongoing studies for this model biofuel-producing strain.

**Results:**

In this study, we determined the complete chromosome and plasmid sequences of ZM4 and its engineered xylose-utilizing derivatives 2032 and 8b. Compared to previously published and revised ZM4 chromosome sequences, the ZM4 chromosome sequence reported here contains 65 nucleotide sequence variations as well as a 2400-bp insertion. Four plasmids were identified in all three strains, with 150 plasmid genes predicted in strain ZM4 and 2032, and 153 plasmid genes predicted in strain 8b due to the insertion of heterologous DNA for expanded substrate utilization. Plasmid genes were then annotated using Blast2GO, InterProScan, and systems biology data analyses, and most genes were found to have apparent orthologs in other organisms or identifiable conserved domains. To verify plasmid gene prediction, RNA-Seq was used to map transcripts and also compare relative gene expression under various growth conditions, including anaerobic and aerobic conditions, or growth in different concentrations of biomass hydrolysates. Overall, plasmid genes were more responsive to varying hydrolysate concentrations than to oxygen availability. Additionally, our results indicated that although all plasmids were present in low copy number (about 1–2 per cell), the copy number of some plasmids varied under specific growth conditions or due to heterologous gene insertion.

**Conclusions:**

The complete genome of ZM4 and two xylose-utilizing derivatives is reported in this study, with an emphasis on identifying and characterizing plasmid genes. Plasmid gene annotation, validation, expression levels at growth conditions of interest, and contribution to host fitness are reported for the first time.

**Electronic supplementary material:**

The online version of this article (10.1186/s13068-018-1116-x) contains supplementary material, which is available to authorized users.

## Background

Advances in next-generation sequencing (NGS) technology, mass spectrometry, and integrative omics approaches have facilitated more detailed studies of microbial responses to environmental and stress conditions. However, integrative analysis of multi-omic datasets requires the availability of complete genome sequences, i.e., chromosomal and extrachromosomal genomic content, in addition to accurate gene identification and functional annotation.

*Zymomonas mobilis* is a natural ethanologen being developed for industrial conversion of biomass feedstocks into biofuels [1, 2]. Unlike the classical ethanologen *Saccharomyces cerevisiae*, which uses the Embden–Meyerhof–Parnas (EMP) pathway for glycolysis, *Z. mobilis* uses the Entner–Doudoroff (ED) pathway, resulting in less ATP produced per molecule of glucose consumed and higher ethanol yield [3]. Furthermore, *Z. mobilis* is tolerant to many inhibitors found in biomass hydrolysates and therefore has the potential to be developed as a platform catalyst using feedstocks from widely different sources [4–6]. Recently, it was demonstrated that *Z. mobilis* can utilize atmospheric N_2_ as the sole nitrogen source during fermentation without affecting ethanol yield, which could significantly reduce the cost of cellulosic ethanol production [7]. Besides ethanol, *Z. mobilis* has recently been engineered to produce 2,3-butanediol, which can serve as a precursor for the generation of advanced biofuels and bioplastics [8].

Phenotype microarray profiling of *Z. mobilis* ZM4 indicates that *Z. mobilis* is closely related to yeast physiologically [9]. Following the first transcriptomic and metabolomic study of *Z. mobilis* during aerobic and anaerobic fermentation [10], transcriptomics has been widely used to study the stress responses of *Z. mobilis* to high glucose concentration, ethanol, furfural, phenolic compounds, and other inhibitors [11–14]. In addition, proteomics studies have provided an in-depth understanding of acetate and ethanol stress responses [15, 16]. With the rapidly decreasing costs of next-generation sequencing (NGS), it is expected that NGS-based systems biology studies will be widely applied in the future. However, despite advances in this direction, improvements in genome sequencing accuracy and annotation are still required to meaningfully interpret microbial physiology via high-throughput omics analyses.

To facilitate multi-omic data analyses, the genomes of eight *Z. mobilis* strains have been completely sequenced, including model strain ZM4 [17–23]. In 2009, we improved the genome annotation of *Z. mobilis* ZM4 (AE008692.2) [24]. We additionally reported the sequence and annotation of five plasmids in ZM4, which were absent in the original ZM4 genome sequence report in 2005 [17]. These plasmids, pZZM401 to pZZM405, were deposited in 2010 under the GenBank Acc. No. CP001881.1–CP001885.1. However, due to a strain identity issue propagated by scientific literature, patent deposits, and bacterial culture collections in both Europe and USA [20, 25], these plasmid sequences were actually derived from strain CP4 instead of the previously reported ZM4. This issue became apparent when (1) CP4 was sequenced to completion in 2013 and proved not to be identical to ZM4 as previously thought [20]; (2) an already sequenced ZM4 41-kb plasmid (plasmid 1; AY057845.1) was not identical to any reported ZM4 or CP4 plasmids, instead each and every newly sequenced CP4 plasmid has high similarity to plasmids from pZZM401 to pZZM405; and (3) NGS reads of ZM4 from both genome resequencing and RNA-Seq studies were unable to map to the majority of the pZZM401 to pZZM405 plasmid sequences.

Because of these discrepancies, we decided to re-sequence *Z. mobilis* ZM4 as well as two engineered xylose-utilizing derivatives 2032 and 8b that are implemented in lignocellulose fermentations. Our foremost aim was to identify and annotate missing ZM4 plasmids and assign potential physiological roles for plasmid genes by evaluating gene expression in a variety of growth conditions and lignocellulosic hydrolysates. Plasmid copy numbers were also examined under different conditions.

## Results and discussion

### Genome sequences of *Z. mobilis* ZM4 and its xylose-utilizing derivatives 2032 and 8b

#### Identification of four plasmids in ZM4 and its derivatives

To re-examine the genome of the *Z. mobilis* model strain ZM4 and obtain an unequivocal view of its plasmids, we combined and cross-compared DNA and RNA sequencing results of wild-type ZM4 and its xylose-utilizing derivatives 2032 and 8b from five different research laboratories in the USA, Greece, and China. The genome of strain 2032 was sequenced at the Great Lakes Bioenergy Research Center (GLBRC). The genomes of both ZM4 and 8b were sequenced in three laboratories (NREL, GLBRC, and Hubei University) in 2011, 2014, and 2016, respectively. Lastly, the ZM4 plasmids were re-sequenced anew from plasmid-only DNA samples at the DOE Joint Genome Institute (JGI) in 2013. The results from each sequencing endeavor were assembled independently using different bioinformatics pipelines.

**Fig. 1:**
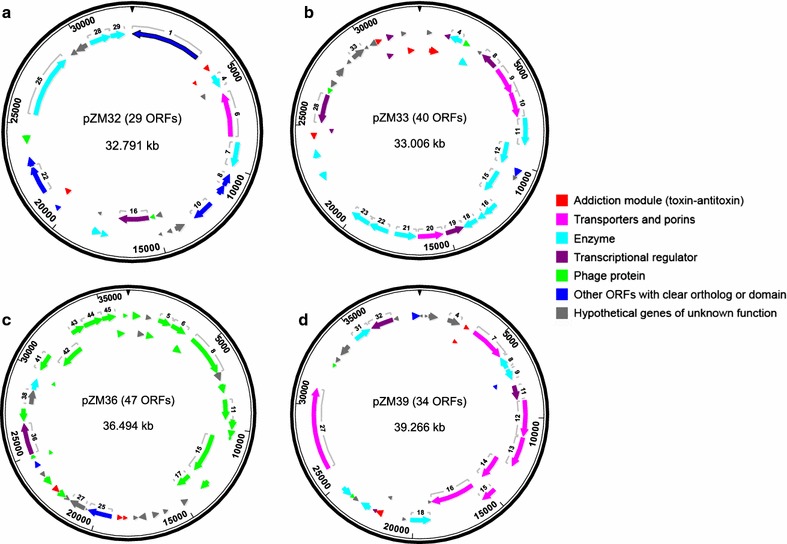
Map of four plasmids in ZM4 and 2032: pZM32 (**a**), pZM33 (**b**), pZM36 (**c**), and pZM39 (**d**). Seven different functional groups of encoding protein are indicated in different colors

These parallel sequencing efforts consistently identified a total of four plasmids in strain ZM4, which were named according to their sequence sizes: pZM32 (32,791 bp), pZM33 (33,006 bp), pZM36 (36,494 bp), and pZM39 (39,266 bp) in ZM4 and 2032 (Fig. 1). Final plasmid sequence assembles were confirmed by primer walking (Additional file 1: Figure S1A), and our results were consistent with previously published gel electrophoresis results in which three plasmids, approximately 32.5, 34, and 40.5 kb in size, were observed in ZM4 [25]. The estimated sizes of these plasmids are very close to the sizes of the four plasmids presented here. The plasmid number discrepancy between gel electrophoresis size estimation and our results is likely due to the fact that pZM32 (32,791 bp) and pZM33 (33,006 bp) are too similar in size to be resolved in an agarose gel and likely migrate together as a single 32.5-kb band. This 32.5-kb band indeed appeared to be more intense in the gel image [25] further supporting the presence of additional DNA migrating at this size. Additionally, our restriction fragment length polymorphism of ZM4 plasmid preparations coincided with previous reports [25].

In strain 8b, sequencing analysis revealed that the P*eno*_*talB*-*tktA*-*cat* cassette was randomly inserted into gene ZMOp41x016 of plasmid pZM36, resulting in plasmid size increase from 36,494 to 40,858 bp. This larger version of pZM36, e.g., the pZM36::P*eno*_*talB*-*tktA*-cat derivative, was named pZM41 (Additional file 2: Table S1). Therefore, all three strains contain pZM32, pZM33, and pZM39, while ZM4 and 2032 possess pZM36 and 8b contains pZM41 with P*eno*_*talB*-*tktA*-cat inserted into pZM36.

pZM39 showed high sequence identity (99%) to the previously published ZM4 plasmid 1 sequence (AY057845). Plasmid 1 is 40,991 bp and appears to contain small portions of other sequences not found in pZM39. By contrast, the four new plasmids reported here show very low sequence identity to the previously published five plasmids (pZZM 401–405) with the exception of a 27-kb region of pZM36 and a 12-kb region of pZM32 with 95–99 and 90% sequence identity to pZZM401 and pZZM405 [24], respectively. pZM33 showed no extensive identity to any previously published ZM4 plasmid sequences. These results confirmed that *Z. mobilis* plasmid 1 sequence in GenBank is derived from ZM4 and that plasmids analyzed and reported herein are new for ZM4.

#### Functional annotation of plasmid genes

As shown in Fig. 1 and Additional file 2: Table S1, a total of 150 genes were identified in all four plasmids across strains ZM4, 2032, and 8b, excluding exogenous genes in the P*eno*_*talB*-*tktA*-cat cassette in pZM41. One hundred and nine plasmid ORFs encode proteins that have either apparent sequence homology to proteins with defined functions in other organisms or identifiable conserved domains. The remaining 41 ORFs match to “hypothetical protein” BLAST hits from other organisms. Most plasmid genes have an ATG start codon, except for rare cases of well-predicted ORFs (i.e., highly homologous to other bacteria) that start with GTG or TTG. Gene ZMOp39x026 (ParA ATPase), which shows high sequence homology to ParA ATPase genes in other *Z. mobilis* strains and members of *Burkholderiales*, was identified as a probable pseudogene as it contains a nonsense mutation at position 24,563. ZMOp33x025 encoding a putative transposase IS4/IS5 family protein was also identified as a probable pseudogene due to an N-terminal frameshift mutation. Lastly, frameshift mutations were found in ZMOp32x029 (Mrr endonuclease), resulting into two separate in-frame ORFs comprising N-terminal and C-terminal regions, respectively, which we combined into a single, but disrupted, ORF (Additional file 2: Table S1).

As shown in Additional file 2: Table S1, several genes involved in endonuclease and other restriction systems were found in pZM32 (ZMOp32x025, ZMOp32x028, and ZMOp32x029). Interestingly, most predicted genes in pZM36 (27 out of 47) are phage-related, encoding phage structure proteins, including tail, head, capsid, and baseplate assembly proteins, and other phage-related functions. All four plasmids encode addiction modules, including toxin-antitoxin systems (DinJ, YafQ, RelB, RelE/ParE, and BrnT), which have been reported to regulate stress adaptation and replicon persistence [26–31]. Genes encoding membrane-associated transporters, symporters, and porins (ABC-transporters, TonB-like, and OprB-like transporters) were also found in the plasmids, especially in pZM39. These efflux pumps have been shown to be functional for carbohydrate uptake or multidrug resistance, and might also play roles in the resistance to cellulosic biomass inhibitors [32–34]. A few transcriptional regulators were also identified in each plasmid. Lastly, several genes, especially those in pZM33, encode enzymes for metabolic functions, such as dehydrogenase, NADPH-dependent oxidoreductase, nucleoside triphosphate hydrolase, amidase, Acyl-CoA *N*-acyltransferase, d-isomer-specific 2-hydroxyacid dehydrogenase, dihydrofolate reductase, pyridoxal phosphate-dependent transferase, and amidohydrolase (Additional file 2: Table S1).

#### Comparisons of chromosomal sequences of ZM4 and its xylose-utilizing derivatives 2032 and 8b

As described in “Methods,” the chromosomal sequence of ZM4, 2032, and 8b were also assembled. The new ZM4 genome reported here is 2058,755 bp, which is 2392 bp larger than the previously published *Z. mobilis* ZM4 genomic sequence (GenBank Acc. No. AE008692.2). The additional sequence consists of a single 2400-bp insertion located in gene ZMO0133, which was confirmed by both PCR and Sanger sequencing using three primer sets covering this region (Additional file 1: Figure S1B). This insertion resulted in the shortening of ZMO0133 from 2493 to 2169 bp (831–723 in amino acid sequence), and added two new ORFs, named ZMO2040 and ZMO2041. Similar to ZMO0133, both of these new ORFs are Sel1 domain protein repeat-containing proteins [35]. RNA-Seq data analysis confirmed the expression of both ZMO2040 and ZMO2041. The expression of ZMO2041, ZMO0133, ZMO0134, ZMO0135, and ZMO0136 was very low in ZM4 when grown in rich medium under both anaerobic and aerobic conditions. However, ZMO2040 was well expressed, but highly variable in rich medium under both anaerobic and aerobic conditions, with highest levels of expression observed at late stationary phase under aerobic conditions.

Resequencing of ZM4 also identified 65 SNPs in both coding and non-coding regions (Table 1) relative to current ZM4 genome release (AE008692.2). Most SNPs are located in non-coding regions or in ribosomal RNA genes (ZMOr002, r005, and r008). We also identified two 1-bp deletions in pseudogene ZM4_P003 (a.k.a. ZMO0983) which results in a 1395-bp continuous ORF encoding an ABC transporter substrate-binding protein. We therefore removed the pseudogene designation and restored this ORF under its initial name ZMO0983. A 7-bp duplicated sequence originally reported in the pseudogene ZMO1617 was found to be absent in all three strains and was confirmed by Sanger sequencing (data not shown). The 7-bp deletion results in a continuous open reading frame for ZMO1617 which we reinstated as a protein-coding gene. In comparison with orthologs from other *Z. mobilis* strains and other bacteria, we also observed that several chromosomal genes have nonsense mutations (stop codons) or frameshift mutations, including ZMO0171, ZMO0955, ZMO1535, and ZMO2036. Lastly, we merged ZMO0168 and ZMO0170 into a single annotation, ZMO0168, encoding a glycerophosphodiester phosphodiesterase (coordinates 151,491–155,014). BLAST analysis revealed that the original ORFs ZMO0168 and ZMO0170 encoded the C-terminal and N-terminal regions of glycerophosphodiester phosphodiesterase, respectively, relative to locus AEH62510 in *Z. mobilis* ATCC 10988. This was likely missed in previous genome annotations as this ORF is disrupted by a large sequence insertion that includes ZMO2000 and ZMO0169. It should be noted here that all new features in the genome sequence of ZM4 were also present in the sequences of 2032 and 8b, and thus were corroborated in triplicate.

**Table 1:** SNPs in *Z. mobilis* ZM4 by comparing new sequence and previous published and modified *Z. mobilis* ZM4 genomic sequence (GenBank: AE008692.2)

Start and end position in ZM4	Location in coding or no-encoding region	Description of SNP in previously published ZM4 sequence (changes from previous one to current one)
122195–122196	ZMO0133 (*hemE*)	Missing 2400 bp fragment. Shorten ZMO0133 from 831 bp to 723 bp, and add two new ORFs (ZMO2040 and ZMO2041)
140833–140857	Promoter of ZMO0152 (*pyk*) and ZMO0153 (*yebC*)	agaaaatcgattttctcgttgggcg to TgTTTatcgaACGGAtTgAACgAcT
140900–140916	Promoter of ZMO0152 (*pyk*) and ZMO0153 (*yebC*)	aaataaaaaacgcacc (16 bp) to CTatTTaGaaGCcaAGT (17 bp)
561507–561515	ZMO0561	ggggggggg to CAACTTTTC (480APPP to 480GKVA)
851609–851609	ZMO0843 (*argS*)	C to T (L471L)
851712–851712	ZMO0843 (*argS*)	C to A (P505H)
973460–973460	ZMO0955/ZM4_P002 pseudogene	T to A
977904–977948	Promoter region of ZMO0961 (*cyc*_C552)	taagatcttatagcagagttctaagtcttaatagatagtttagct to GaaAatTttatagcagagttTtTagGcttTatagCtaTtttGAcG.
1004680–1004681	ZMO0983/ZM4_P003 pseudogene	Extra A in previously published ZM4 sequence
1004686–1004687	ZMO0983/ZM4_P003 pseudogene	Extra T in previously published ZM4 sequence
1614974–1614974	Downstream of ZMO1579 and downstream of ZMOt037	G to A
1615565–1615565	ZMOr002	G to A
1659861–1659867	ZMO1617	Extra cagtttc in previously published ZM4 sequence
1900714–1900714	ZMOr005	G to A
1913696–1913701	Downstream of ZMO1864 and downstream of ZMOt046	cttatt to TttatC
1914566–1914566	ZMOr008	G to A
2058155–2058155	Promoter region of ZMO1998 and ZMO0001	T to C

**Fig. 2:**
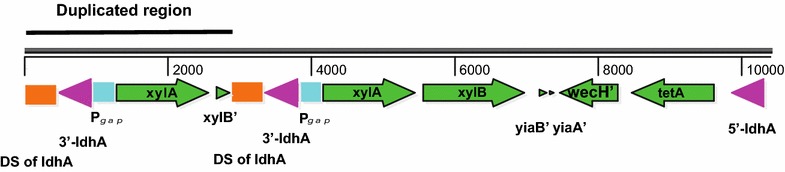
Map of *xylAB*′ duplicated region in the chromosome of *Z. mobilis* 2032. P*gap_xylA*-*xylB*-*yiaB′*-*yiaA′*-*wecH′*-*tetA* (genes are in green color and P*gap* is in blue color) was inserted into ZMO1237 (*ldhA*) in 2032. The orange rectangle indicates downstream (DS) region of *ldhA* and purple arrow indicates either 5′ or 3′ of *ldhA*. Duplicated P*gap*_*xylA xylB′* and portion of 3′ end of *ldhA* and its downstream region is indicated as a line above

The chromosomes of 2032 and 8b are 2072,170 and 2064,754 bp in size, respectively, and are 13,415 and 5999 bp larger than the revised ZM4 chromosome reported in this work. Both 2032 and 8b also have the 2400-bp insertion found in the new ZM4 sequence. Sequencing results confirmed that both strains bear insertions of the exogenous genes that were used to engineer xylose metabolism [36]. In both 2032 and 8b, the 6010-bp P*gap_xylA*-*xylB*-*yiaB*′-*yiaA′*-*wecH′*-*tetA* cassette was found to be inserted at the 3′ end of ZMO1237 (*ldhA*). Interestingly in 2032, integration of P*gap_xylA*-*xylB*-*yiaB′*-*yiaA′*-*wecH′*-*tetA* led to a duplication of *xylA* and partial duplication of *xylB* and ZMO1237 (Table 2, Fig. 2) which was also confirmed by PCR and Sanger sequencing (data not shown). The P*gap_talB*-*tktA*-*cat* cassette that further enables C5-sugar metabolism was found to be chromosomally inserted in strain 2032 into the promoter region between two divergent hypothetical protein-coding genes, ZMO1268 and ZMO2012, while P*eno_talB*-*tktA*-*cat* was inserted in strain 8b into gene ZMOp41x016 of plasmid pZM41, as described above (Tables 2 and 3).

**Table 2:** SNPs in *Z. mobilis* 2032 by comparing with new *Z. mobilis* ZM4 genome sequence

Start and end position in ZM4	Location in coding or no-encoding region	Description of SNP (changes from ZM4 to 2032)
995001–995001	ZMO0976	Insertion one “A,” and frame-shift and shorten ZMO0976 from 1023 to 708 bp (in both 2032 and 8b)
1015532–1015532	Promoter of ZMO0998	C to T
1263984–1265916	ZMO1237 (*ldhA*)	11 bp (CATGGGTCATA) deletion and 2881-bp duplicated Pgap-xylA xylB′ inserted into ZMO1237 (ldhA) 3′-end, with ZMO1237 shorten from 996 bp to 498 bp
1265917–1266864	ZMO1237 (*ldhA*)	Duplicated downstream and 3′ end of ZMO1237 sequence
1266865–1272874	ZMO1237 (*ldhA*)	6010 bp Pgap-xylA xylB yiaB yiaA wecH tetA cassette inserted into ZMO1237
1272889–1272889	ZMO1237 (*ldhA*)	G to A (S157S) (in both 2032 and 8b)
1295154–1299678	Promoter region of ZMO1268 and ZMO2012	4525 bp Tn5 cat Pgap-talB tktA inserted between ZMO1268 and ZMO2012
1299679–1299687	Promoter region of ZMO1268 and ZMO2012	Duplicated 9 bp ttcctagat (same sequence at 1295145–1295153)
2007353–2007353	ZMO1934	C to T (E50K) (in both 2032 and 8b)

**Table 3:** SNPs in *Z. mobilis* 8b by comparing with new *Z. mobilis* ZM4 genome sequence

Start and end position	Location in coding or no-encoding region	Description of SNP (changes from ZM4 to 8b)
95726–95726	ZMO0109	C to T (C150C)
131578–131578	ZMO0143	C to T (L55F)
508460–508460	ZMO0505 (*rluC*)	G to A (G165G)
511672–511672	ZMO0510 (*pdhC*)	G to A (G81S)
512937–512937	ZMO0511	G to A (V63I)
519279–519279	ZMO0516	G to A (V142I)
529169–529169	ZMO0533 (*rpsE*)	G to A (S198N)
529994–529994	ZMO0536 (*rplO*)	G to A (V128I)
532460–532460	ZMO0538 (*adk*)	G to A (G217D)
537400–537400	ZMO0543 (*acnA*)	G to A (G433D)
569817–569817	ZMO0566	G to A (P13S)
625382–625382	Promoter of ZMO0629 (*fliC*)	C to T
630791–630791	ZMO0636	C to T (P42S)
643884–643884	ZMO0653	A to G (I148I)
658560–658560	ZMO0663 (*radC*)	G to A (L174L)
674692–674692	ZMO0680	G to A (P276P)
691579–691579	ZMO0692 (*gyrA*)	G to A (N220N)
698481–698481	Promoter of ZMOt012	G to A
716406–716406	ZMO0715 (*aspS*)	C to T (L544L)
743017–743017	ZMO0743 (*prfC*)	C to T (T136I)
846559–846559	ZMO0840	G to A (R350R)
849934–849934	ZMO0842 (*dgtP*)	G to A (G366D)
905206–905206	ZMO0893	C to T (A14T)
916878–916878	ZMO0904 (*bga*)	C to T (G760D)
950749–950749	ZMO0927 (*aat*)	G to A (L125L)
995001–995001	ZMO0976	Insertion one “A,” and frame-shift and shorten ZMO0976 from 1023 to 708 bp (in both 2032 and 8b)
1019918–1019918	Promoter of ZMO1001	C to T
1044279–1044279	ZMO1025 (*nrdD*)	C to T (T509I)
1051161–1051161	ZMO1036 (*argG*)	C to T (L46F)
1079284–1079284	ZMO1064	C to T (L11F)
1130189–1130189	ZMO1113 (*ndh*)	G to A (V239I)
1159680–1159680	ZMO1139 (*ilvI*)	C to T (S524F)
1176064–1176064	ZMO1156 (rpsB)	C to T (R97H)
1179870–1179870	ZMO1162	C to T (A94 V)
1250454–1250454	Promoter of ZMO1223 (*fabD*) and ZMO1225 (*rpsF*)	C to T
1262326–1262326	ZMO1236 (*adhA*)	C to T (A153T)
1263377–1263378	Promoter of ZMO1236 (*adhA*) and downstream of ZMO1237 (*ldhA*)	Missing one base “C”
1263983–1269992	ZMO1237 (*ldhA*)	11 bp (CATGGGTCATA) deletion and 6010 bp Pgap-xylA xylB yiaB yiaA wecH tetA inserted into ZMO1237 3′-end, with ZMO1237 shorten from 996 to 498 bp
1270007–1270007	ZMO1237 (*ldhA*)	G to A (in both 2032 and 8b)
1330074–1330074	ZMO1307 (*fumA*)	C to T (T69I)
1363705–1363705	ZMO1341	C to T (G248D)
1367432–1367432	ZMO1345 (pepN)	C to T (A493T)
1397162–1397162	ZMO1377	G to A (P447S)
1412666–1412666	ZMO1390	G to A (L243F)
1417484–1417484	ZMO1395 (*hutG*)	G to A (A257V)
1430724–1430724	ZMO1409 (cbbF)	G to A (G220G)
1442443–1442443	ZMO1422	G to A (I400I)
1447932–1447932	ZMO1424 (*clpB*)	G to A (V374I)
1461618–1461618	ZMO1435 (leuS)	G to A (H13Y)
1488113–1488113	ZMO1461	G to A (L174F)
1498927–1498927	ZMO1470	G to A (A277V)
1499416–1499416	ZMO1470	G to A (P114L)
1509299–1509299	ZMO1479 (*etfA*)	G to A (A48V)
1519583–1519583	ZMO1485	C to T (D284D)
1542900–1542900	ZMO1511	G to A (R130K)
1572765–1572765	ZMO1535	C to T (S9F)
1585059–1585059	ZMO1544 (*cobS*)	C to T (A263T)
1639561–1639561	ZMO1592 (*alr*)	A to G (Y92Y)
1698229–1698229	ZMO1646	G to A (E364 K)
1713092–1713092	ZMO1657	G to A (K176K)
1715028–1715028	ZMO1659 (*ftsH*)	G to A (V68I)
1739226–1739226	ZMO1685 (*serA*)	A to G (T93A)
1890021–1890021	ZMO1842 (*nosX*)	C to T (G277D)
1894604–1894604	ZMO1848	C to T (K347K)
1917360–1917360	ZMO1862	C to T (A33T)
1945390–1945390	ZMO1886	C to T (V343I)
1999937–1999937	ZMO1934	C to T (E50K) (in both 2032 and 8b)
2018321–2018321	ZMO1955 (*yqkJ*)	G to A (S388F)

Both 2032 and 8b have all 65 SNPs identified from the new ZM4 sequence (Table 1). They also have additional sequence variations not present in strain ZM4 (Tables 2 and 3), including a synonymous SNP in ZMO1237, which encodes d-isomer-specific 2-hydroxyacid dehydrogenase NAD-binding protein and a non-synonymous SNP in ZMO1934 resulting in an E50K substitution of N-6 DNA methylase. Most *Z. mobilis* strains have a glutamate residue at position 50 of ZMO1934. Both strains also have a single adenosine insertion in ZMO0976 (xylulose reductase), resulting in a frameshift at codon 211 and a premature stop codon at 236, which might beneficially impact xylose utilization through reduced xylitol production if the premature stop codon results in a reduced or loss of function protein [37]. We also identified 64 SNPs, within coding and non-coding regions, that are unique to strain 8b when compared to 2032 and ZM4 (Table 3), which could have been generated during the chemical mutagenesis and directed evolution performed on 8b to increase xylose utilization efficiency after incorporation of *E. coli* xylose-metabolizing genes.

In addition to the genome sequence revisions described here, we updated all chromosomal gene annotations based on data compiled from new NCBI-blast and InterProScan searches, previous ZM4 GenBank submissions, and Microbes Online (www.microbesonline.org). All chromosomal sequences and annotation revisions for strains ZM4, 2032, and 8b are available at GenBank under the following accession number: CP023715-9 for ZM4 chromosome, pZM32, pZM33, pZM36, and pZM39, respectively; CP023677-81 for 2032 chromosome, pZM32, pZM33, pZM36, and pZM39, respectively; and CP023682-6 for 8b chromosome, pZM32, pZM33, pZM39, and pZM41, respectively.

### Evaluation of plasmid copy number changes during anaerobic and aerobic growth

**Fig. 3:**
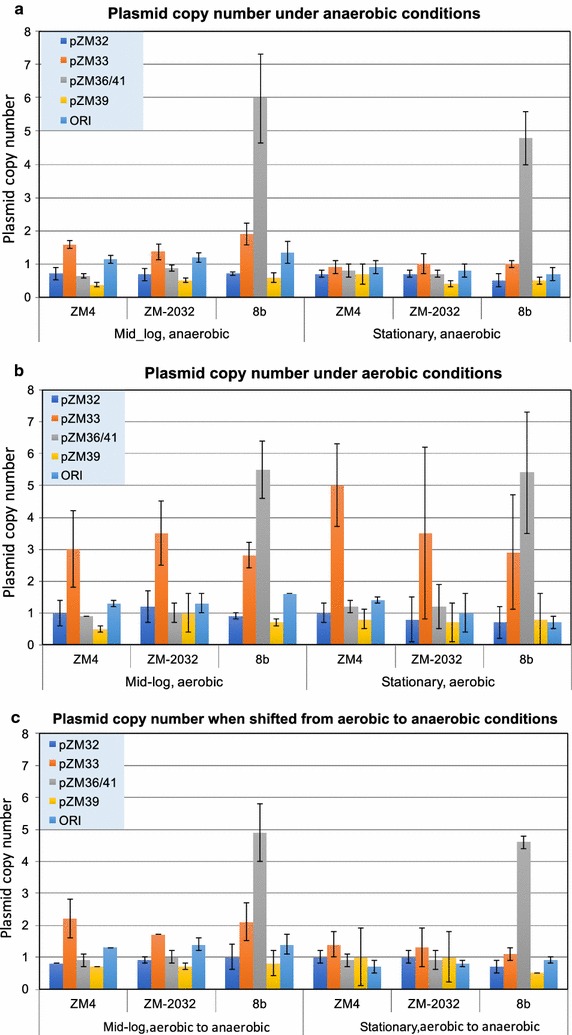
Plasmid copy numbers in *Z. mobilis* ZM4, 2032, and 8b under anaerobic conditions (**a**), aerobic conditions (**b**), and shift from aerobic to anaerobic conditions (**c**). All *Z. mobilis* strains were grown in RMG medium under anaerobic, aerobic or shift from aerobic-to-anaerobic conditions, and plasmid copy numbers were measured at both Mid-log and Stationary phases relative to the chromosome terminus assigned as 1 and corrected for PCR efficiency (see “Methods”). The chromosome *ori* copy number was determined as a control. Plasmid copy number measurements were based on at least three biological replicates, with the standard deviation indicated as error bar

To characterize the newly identified ZM4 plasmids, and how they may broadly impact host biology, we examined plasmid copy numbers in cells grown in rich media containing 2% glucose under anaerobic and aerobic conditions (Fig. 3a, b) and monitored plasmid copy numbers when cells were shifted from aerobic to anaerobic conditions (Fig. 3c). Using calibrator plasmids grown in *E. coli* (see “Methods” and Additional file 3: Table S2) as a reference, pZM32, pZM36, and pZM39 showed similarly low copy numbers (~ 1 copy/cell) in all strains under anaerobic conditions, with pZM33 showing a slightly higher copy number during exponential growth. pZM41 in 8b showed markedly elevated copy numbers under anaerobic conditions, with six and five copies at mid-log and stationary phases, respectively (Fig. 3a). In aerobic conditions, pZM32, pZM36, and pZM39 again showed similarly low copy numbers and pZM33 was again higher, showing as much as 5–6 times more copies relative to other plasmids under aerobic growth (Fig. 3b). Interestingly, pZM33 was ~two- to sixfolds higher in aerobic conditions compared to anaerobic conditions at both mid-log and stationary phases, which may indicate a role for pZM33 genes in mediating aerobic growth. pZM41 in 8b was also elevated to over five copies under aerobic conditions, and slightly increased in copy number as growth progressed. The parental copy of pZM41, pZM36 in ZM4 and 2032, did not change considerably in aerobic conditions, suggesting that elevated copy number is a distinct feature of pZM41 in strain 8b. When cells were shifted from aerobic to anaerobic conditions, plasmid copy numbers closely resembled anaerobic growth levels, with pZM33 somewhat retaining its elevated copy number and pZM41 remaining of considerably higher copy number than the parental pZM36 and remaining plasmids (Fig. 3c).

The dramatic increase in the copy number of pZM41 compared to that of the parental pZM36 might be related to the insertion of P*eno_talB*-*tktA*-*cat*. Beside the effect of the insertion on backbone plasmid features and on the knock-out of the affected gene (a putative phage tail assembly gene), another possibility is that either transketolase (*tktA*), transaldolase (*talB*) alone or both are beneficial to *Z. mobilis*, possibly via increased production of d-ribose-5-phosphate for nucleic acid biosynthesis and d-erythrose-4-phosphate for aromatic amino acids biosynthesis (shikimate pathway). Although *Z. mobilis* is not a native pentose-fermenting organism, there is a transketolase (ZMO0176) encoded in the chromosome of ZM4, suggesting that transketolase might be an important enzyme for metabolic function beyond pentose utilization in *Z. mobilis*. Enzymes of the pentose phosphate pathway have been found in some other non-pentose metabolizing organisms, including *S. cerevisiae*, which metabolize glucose for amino acid and nucleic acid synthesis [38].

These results indicate that ZM4 native plasmids have very low copy numbers under anaerobic conditions, which increase mildly under aerobic conditions in all strains tested. In contrast, the copy number of pZM41 (pZM36::P*eno_talB*-*tktA*-*cat*) could be manifesting a five- to sixfold increase in 8b compared to the parental pZM36 in ZM4, while the copy number of pZM33 also seemed to increase moderately under aerobic conditions.

### Verification of plasmid gene expression through omic and physiological studies

#### Evaluation of rRNA depletion approaches to enrich mRNA for RNA-Seq

To assess the function of plasmid genes in response to environmental conditions, we analyzed gene expression (RNA-Seq) data from ZM4, 2032, and 8b grown in rich media containing glucose as a carbon source, as well as in biomass hydrolysates at the logarithmic or stationary stage (see RNA-Seq metadata in Additional file 4: Table S3). Data from 45 RNA-Seq samples with 2–5 biological replicates for each condition were included for analyses (Additional file 4: Table S3). The ORNL/NREL group tested several rRNA depletion methods/kits for rRNA depletion in *Z. mobilis* since 2009, which were inefficient at that time due to poor matches to probe sequences used for subtractive hybridization. A customized *Zymomonas* rRNA depletion kit was then developed with Life Technologies and verified by RT-qPCR. The new kit reduced rRNA content from 90% or more of the total RNA to less than 8.5% for several strains grown under different conditions (Additional file 1: Figure S2A–C). After rRNA depletion with this customized kit, samples showed good correlation with a coefficient R-square value in a range of 0.96–0.98 (Additional file 1: Figure S2D). The GLBRC group likewise tested several rRNA depletion methods/kits for rRNA depletion in *Z. mobilis* and found that the Ribo-Zero rRNA Removal Kit from Epicentre/Illumina was the most effective, removing up to 90% rRNA. This same kit was able to remove about 50% of the rRNA for samples processed at JGI, where RNA-Seq was performed for GLBRC and the NKUA.

#### Multivariate analysis of RNA-Seq data

To broadly assess transcriptomic differences in our RNA-Seq data, we clustered the expression of all 150 plasmid genes across a range of growth conditions and growth phases obtained by our three research groups (NKUA, GLBRC, and NREL). To avoid both batch effects associated with these independent sets of data generated from three different locations and the possible influence of chromosomal responses after genome-wide normalization, RNA-Seq data were normalized in the following way: (1) reads mapped to plasmid genes were isolated; (2) FPKM (fragments per kilobase per million mapped reads) values were computed specifically for the plasmid set; and (3) the plasmid-specific FPKM values were additionally quantile-normalized.

**Fig. 4:**
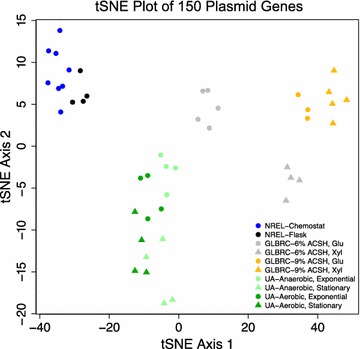
t-SNE analysis of the plasmid-restricted expression data from the 3 laboratories. Blue, NREL, fermentor with biomass hydrolysates; black, NREL, flasks with rich RMG medium; light gray, GLBRC, 6% ACSH; Orange, GLBRC, 9% ACSH; light green, Univ. Athens (UA), anaerobic; dark green, UA, aerobic. Circles, exponential growth stage (exponential in UA dataset and Glucose stage, Glu, in GLBRC dataset); triangles, later growth stage (stationary stage in UA dataset and xylose stage, Xyl, in GLBRC dataset)

Normalized FPKM values were used for global structure identification with t-distributed stochastic neighbor embedding (t-SNE) algorithm (Fig. 4). The t-SNE algorithm is a viable alternative to principal component analysis (PCA) to detect main trends in omic data [39, 40], particularly when analyzing fewer data points. Based on the results of Fig. 4, stationary growth stage was separated by having a higher value of t-SNE mapping along Axis 1 for each dataset that included samples from both exponential and stationary growth stages (NKUA and GLBRC). Moreover, different glucan-loading (6% vs. 9%) AFEX-corn stover hydrolysates (ACSH) in the GLBRC dataset and, to lesser extent, different oxygen concentrations (anaerobic vs. aerobic) in the NKUA dataset, or fermenter (biomass hydrolysate) and flask experiments (rich medium with glucose) in NREL dataset, may be separated using the expression information for 150 plasmid genes exclusively.

Interestingly, when cells were grown in hydrolysate from higher biomass loading (i.e., 9% glucan loading), this appeared to shift exponential growth phase (glucose stage) plasmid gene expression patterns towards an expression state more similar to that of stationary growth phase (xylose stage) in low biomass loading (i.e., 6% glucan loading). This phenomenon was visible in t-SNE analysis (Fig. 4, large right-shift along the first t-SNE axis of both 9% ACSH samples and 6% ACSH samples at Glucose stage), hierarchical clustering (Additional file 1: Figure S3, high closeness of expression signatures of 6% ACSH at xylose stage and 9% ASCH at glucose stage), and global correlation screening across all RNA-Seq libraries (data not shown).

#### Verification of plasmid gene expression through RNA-Seq analysis

To compare plasmid gene expression under various growth conditions, we performed fermentations with *Z. mobilis* under anaerobic and aerobic conditions, as well as in different lignocellulosic hydrolysates (see “Methods,” and RNA-Seq metadata in Additional file 4: Table S3). Plasmid gene expression data are shown in Additional file 5: Table S4. We chose two datasets for further analysis: anaerobic vs. aerobic for strain ZM4, and 6% vs. 9% glucan loading ACSH for strain 2032, focusing on exponential time point samples only.

Hierarchical clustering of plasmid gene expression showed distinct clustering by growth conditions, with biological replicates clustered together (Additional file 1: Figure S3 and Additional file 6: Table S5). Anaerobic and aerobic conditions were more related to one another than ACSH samples. As shown in Additional file 2: Table S1, Additional file 7: Table S6 and Additional file 8: Table S7, among genes that show statistical significance of differential expression (see “Methods”), 65 genes were either up-regulated or down-regulated by more than twofold in 9% ACSH compared to those in 6% ACSH, while only 11 genes showed greater than twofold changes between aerobic and anaerobic conditions. Among those 11 genes, only three genes from plasmids pZM32, pZM33, and pZM39 showed consistent differential expression at a minimum cutoff value of 1.5-fold change (considering all replicate libraries in two groups), or critical coefficient [41]. Given the greater number of dysregulated plasmid genes in highly concentrated ACSH, these results suggest that plasmid genes may have an important role in mediating fermentation of lignocellulosic hydrolysates. Conversely, our results suggest that plasmid genes may have a more limited role for conventional, rich media growth in both anaerobic and aerobic conditions during exponential growth.

While a considerable number of microarray-based transcriptomic studies have been performed with *Z. mobilis*, to the best of our knowledge, this is the first report of RNA-Seq transcriptional profiling in *Z. mobilis*. RNA-Seq technology offers a number of advantages compared to microarrays especially broad linear range, cross comparison, and higher sensitivity in transcript recognition. The assessment of rRNA depletion methods reported here as well as the pipeline of RNA-Seq data analysis and resulting data sets, will benefit future RNA-Seq transcriptomic studies in this model ethanologenic bacterium.

#### Proteomic and phenotypic analysis of plasmid genes

To further validate and characterize *Z. mobilis* ZM4 plasmid genes, proteomic mass spectrometry data were collected for ZM4 in anaerobic and aerobic fermentation in rich and minimal media at GLBRC independently of the aforementioned RNA-Seq sample collection. We also analyzed proteomic data for ZM4 in ethanol shock and sodium acetate stress conditions at two different time points reported previously [15, 16]. Proteomic data analysis in ethanol shock and sodium acetate stress conditions identified 48 plasmid protein peptides, including phage proteins, membrane proteins, transporters, and toxin-antitoxin systems (Additional file 2: Table S1 and Additional file 9: Table S8). Seventy-one plasmid protein peptides (about 47% of total plasmid ORFs) were identified under anaerobic and aerobic fermentation conditions (Additional file 2: Table S1 and Additional file 10: Table S9), including transporters, toxin-antitoxin systems, and transcriptional regulators. Among them, 40 plasmid protein peptides were detected independently in both research institutes. A total of 79 plasmid encoded proteins were detected across all proteomic data sets that we analyzed, while another 40 plasmid encoded proteins were detected in at least one experiment.

Previously, Deutschbauer et al. [42] used a chemical genomic approach to assay mutant phenotypes using a barcoded transposon library of *Z. mobilis* ZM4. Transposon insertions were mapped to the then published pZZM4015 to pZZM405 plasmid sequences, identifying transposons in 60 plasmid genes. As we have demonstrated in this study, plasmids pZZM401-405 did not originate from *Z. mobilis* strain ZM4, suggesting that plasmid transposon insertions, and subsequent phenotypes, were misattributed to pZZM401-405 genes. We extrapolated the findings of Deutschbauer et al. to identify possible mutant phenotypes for plasmid genes identified in our study to more accurately reflect possible functions of plasmid genes in *Z. mobilis* ZM4. Using BLAST, we identified sequence homologs between genes from previous mutant phenotypic studies and genes identified in pZM32, pZM33, pZM36, and pZM39 (Additional file 2: Table S1). In doing so, we assigned 54 mutant phenotypes to *Z. mobilis* ZM4 plasmid genes (Additional file 2: Table S1). Because of incorrect ZM4 plasmid sequences, past transposon libraries screens missed many actual ZM4 plasmid genes, underscoring the impact that our findings will have on future screens of this kinds. Our RNA-Seq work suggests a role for plasmid genes during biomass hydrolysate fermentation, and further work should be taken to identify mutant phenotypes for all ZM4 plasmid genes through the construction of new DNA-barcoded mutant libraries.

#### Comparison of growth and sugar utilization of *Z. mobilis* 2032 in differently concentrated corn stover hydrolysates (6 and 9% ACSH)

**Fig. 5:**
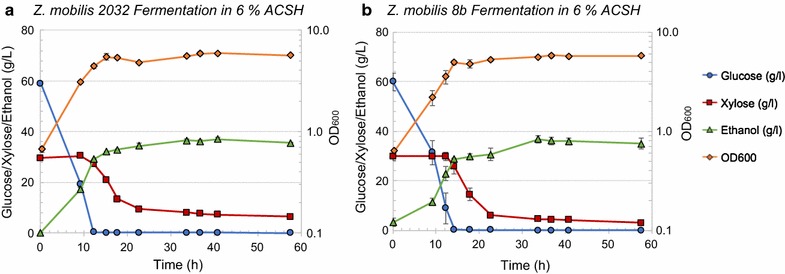
Comparative fermentation of *Z. mobilis* 2032 (**a**) and 8b (**b**) in 6% ACSH. Left axis: glucose (circle), xylose (square), and ethanol (triangle) concentration in the bioreactors (g/L). Right axis: OD_600 nm_ for cell growth (diamond)

**Fig. 6:**
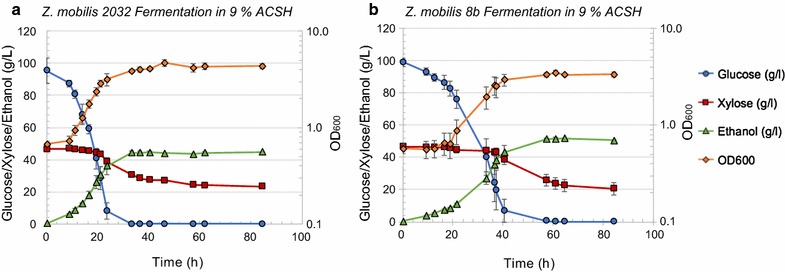
Comparative fermentation of *Z. mobilis* 2032 (**a**) and 8b (**b**) in 9% ACSH. Left axis: glucose (circle), xylose (square), and ethanol (triangle) concentration in the bioreactors (g/L). Right axis: OD_600 nm_ for cell growth (diamond)

Comparative fermentations of each strain in both 6% and 9% ACSH (Figs. 5 and 6). Consistent with previous reports [4], 2032 grew well in 6% ACSH without any supplementation and consumed glucose completely and ~ 20 g/L of the xylose within 40 h. Comparatively, and as shown in Fig. 5, 8b showed marginally slower growth and glucose utilization in 6% ACSH, but utilized slightly more xylose (~ 3 g/L xylose left) than 2032 (~ 7 g/L xylose left). In this respect, the observed trend of 8b to outperform 2032 in RMGX was repeated in 6% ACSH. However, the process ethanol yields were very similar in both strains: 80 ± 1 and 78 ± 2% for 2032 and 8b in 6% ACSH, respectively.

When 2032 and 8b were grown in highly concentrated 9% ACSH, the lag phase was shorter for 2032 (10 h) compared to 8b (~ 20 h) (Fig. 6). Consequently, 8b required more time to completely consume glucose than 2032. The inhibition of cell growth for strain 8b in 9% ACSH might be caused by greater sensitivity to higher osmolality or higher concentrations of inhibitors such as lignocellulose-degradation products. In contrast to fermentation of 6% ACSH, both 8b and 2032 consumed only small amounts of xylose when glucose was nearly depleted but failed to consume additional xylose once glucose was completely depleted (Fig. 6). The process ethanol yields were 69 ± 2 and 59 ± 1% for 2032 and 8b in 9% ACSH, respectively. Further experiments are needed to elucidate the mechanism(s) of poor xylose utilization by *Z. mobilis* in highly concentrated ACSH hydrolysates.

Although both 8b and 2032 were engineered to metabolize xylose by the introduction of two separate expression cassettes, a different promoter of *Peno* was used to control the expression of *talB* and *tktA* in 8b relative to the *Pgap* promoter used to control the expression of these genes in 2032. Differences in the activity and regulation of these two promoters, P*eno* and P*gap* in 8b and 2032, respectively, may likewise affect the expression of *talB* and *tktA* and consequently the growth on xylose. Likewise, differences in gene amplifications in 2032, e.g., duplication of *xylA*, and 8b, e.g., increased copy number of the pZM41 plasmid that carries the *talB* and *tktA* genes (Fig. 3a, b), may contribute to observed phenotypic differences. Additional genetic differences identified between these two strains (Table 3), e.g., mutations in transport and membrane proteins ZMO0143, ZMO0566, ZMO1659, and ZMO1886, could facilitate differences in xylose transportation and utilization, possibly at the expense of lignocellulosic-derived inhibitor uptake. Additional studies are needed to investigate if and how such factors may account for differences observed between 8b and 2032 performance in ACSH and the previously reported superiority of 8b in assimilating xylose in RMGX medium.

## Conclusions

In summary, the genome sequences including complete chromosomal and plasmid sequences in the model ethanologen *Z. mobilis* strain ZM4 and its xylose-utilizing derivatives of 2032 and 8b have been determined and characterized with their possible contribution to cell fitness inferred through a joint effort from four laboratories in three countries. Resequencing of the ZM4 chromosome identified 65 SNPs and a 2400-bp insertion relative to previously published ZM4 chromosomal sequence (AE008692.2). These sequence variants were present across all strains, and additional strain-specific SNPs were identified, especially in strain 8b which had undergone strain adaptation to improve xylose utilization capability. Our findings also suggest that the discovered genetic differences between two xylose-utilizing strains may be pertinent to the observed differences in fermentation performance and hint to engineering strategies that can be adopted during strain development for biofuel production.

Moreover, four native plasmids were identified in all strains, ranging in size from 32 to 39 kb, and harboring in total 150 predicted ORFs. Plasmid sequences across all three strains are identical except for plasmid pZM36, whose allelic replicon in 8b (pZM41) bears an insertion of the heterologous genes *talB*, *tktA*, and *cat*. Plasmid genes were further functionally annotated and characterized through transcriptomic and proteomic studies as well as results from published mutant phenotypic screens. Our results also indicate that all plasmids are present at relatively low copy number except for plasmid pZM33, whose copy number detectably increased in aerated cultures. Plasmid copy number could be affected by growth conditions and heterologous gene insertion in pZM41.

Our findings also suggest that the discovered genetic differences between two xylose-utilizing strains may be pertinent to the observed differences in fermentation performance and hint to engineering strategies for strain development. The revision of the genome of the model ethanologenic *Z. mobilis* strain ZM4 and its xylose-utilizing derivatives of 2032 and 8b will also facilitate future systems biology studies for strains in the ZM4 lineage and could stand as a standard for other *Zymomonas* strains as well.

## Methods

### Strains and growth media

Three *Z. mobilis* strains, the wild-type ZM4 (ATCC31821), and its xylose-utilizing derivatives 2032 (ATCC31821-5C P*gap_taltkt*/P*gapxylAB*, PTA-6977) and 8b (ATCC31821-5C P*eno*_*taltkt*/P*gapxylAB*, PTA-6976), were obtained from American Type Culture Collection (ATCC) or from NREL. Both 2032 and 8b were engineered at the National Renewable Energy Laboratory (NREL) for xylose utilization by introducing *Escherichia coli xylA*, *xylB*, *talB*, and *tktA* genes into *Z. mobilis* ZM4 by either homologous recombination or Tn5 transposon insertion [36]. All strains were grown in rich media (RM) [43] with glucose (RMG): 10 g/L yeast extract, 2 g/L KH_2_PO_4_, 20 g/L glucose or different glucose concentrations as indicated.

### Biomass hydrolysate production

#### NREL hydrolysate preparation

##### Deacetylation of corn stover (P120927DCS)

Corn stover provided by Idaho National Labs (INL) Lot #3 was used for the preparation of deacetylated corn stover used in this study. Deacetylation was performed at 8% (w/w) total solids (TS) concentration with 1500 kg total mass at 80 °C, 2 h, and 0.4% (w/w) NaOH in the dynamic impregnator (DI) vessel at NREL. The DI was mixed at 15 rpm during deacetylation. After deacetylation, the spent caustic liquor was drained from the vessel, leaving the remaining solids at 12% TS. The remaining solids were then rinsed with 950 kg of water, which was drained from the vessel and discarded.

##### Acid impregnation of corn stover (P120927CS)

For the non-deacetylated material (P120927CS), corn stover (INL Lot #3) was added at 8% (w/w) TS with 1500 kg total mass into the DI. The corn stover was soaked in a 0.8% (w/w) sulfuric acid solution for 2 h, then dewatered to 45–50% (w/w) TS prior to pretreatment using a Vincent model CP-10 screw press (Tampa, FL). For the deacetylated material (P120927DCS), water and sulfuric acid were added to the rinsed solids to achieve 8% (w/w) TS and 0.8% (w/w) sulfuric acid in the DI. This slurry was mixed at 15 rpm for two hours prior to dewatering to 45–50% (w/w) TS using the Vincent screw press (Tampa, FL) prior to pretreatment.

##### Pretreatment of corn stover

The corn stover feedstock used to feed the Metso 1-ton/day pretreatment reactor was INL Lot 5, which was knifed milled through a 3/4″ screen and pretreated at 160 °C for 10 min with the residence time based on the assumption of plug flow in the reactor. Pretreatment of deacetylated and non-deacetylated corn stover was performed in the 1-ton/day continuous horizontal pretreatment reactor. Pretreated corn stover was stored at 4 °C prior to further processing.

##### Enzymatic hydrolysis (EH)

Pretreated corn stover (PCS) lots, P120927CS/PCS-01 Drum #1 and P120927DCS/DCS-02 (deacetylated) Drum #1 were neutralized to pH 5.3 using 30% ammonium hydroxide (NH_4_OH, 29.8% assayed as NH_3_, J.T. Baker, Phillipsburg, NJ). The substrate was mixed using a Kitchen Aid mechanical mixer. For each lot of PCS, 2 kg of material was placed in the mixing bowl. Lot P120927CS/PCS-01 had an initial pH of 1.72 and required 32 mL of 30% NH_4_OH to reach a final pH of 5.33. Lot P120927DCS/DCS-02 had an initial pH of 1.66 and required 19 mL of 30% NH_4_OH to reach a final pH of 5.29. The pH-adjusted PCS was stored at 4 °C overnight. Novozymes CTec2 (Lot #VCPI0007) was added to the neutralized slurry supplemented with sterile DI water to constitute 20% total solids at a loading of 40 mg protein/g cellulose for saccharification (48 °C, 150 rpm shaking). After 120 h, the saccharified material was centrifuged at 10,000 rpm using a Sorvall Evolution R centrifuge for 10 min, and then filter sterilized using 0.2 µm Nalgene filters.

#### 6 and 9% glucan-loading AFEX-corn stover hydrolysate production at GLBRC

6% glucan-loading corn stover hydrolysate (6% ACSH) was produced from AFEX-pretreated feedstocks using enzymatic hydrolysis methods as described previously [4]. For making highly concentrated 9% glucan-loading corn stover hydrolysate (9% ACSH), we separated single biomass and enzyme loading used for 6% ACSH into three loadings: 5, 2, and 2%. The first loading of biomass was added into the 15-L vessel of Applikon *ez*-control bioreactor system (Applikon Biotechnology, Foster City, CA) with water, and the entire vessel was autoclaved for 2 h at 121 °C. After cooling to ~ 50 °C, undiluted HCl was added to decrease the initial pH and optimize enzyme activity, and then mixed into the slurry by hand shaking the vessel. First loading of CTec2 and HTec2 from Novozymes (Franklinton, NC) was added. After the hydrolysis was carried out at 50 °C for overnight (~ 16–18 h), the second 2% biomass and enzyme loading were added. Since the biomass in the second loading is not autoclaved, we added tetracycline (final concentration at 6.25 µg/mL) to control contamination observed before [4]. After hydrolysis for another 6 h, the third 2% biomass and enzyme loading were added. The hydrolysis was carried out at 50 °C for 5 days with a stirring speed of 700 rpm. We also increased total enzyme loading by 1.5-fold compared to that used for 6% ACSH.

### Growth and fermentation conditions

Anaerobic and aerobic flask growth conditions for collecting RNA samples have been previously described [44]. Aerated cultures are shaken at 200 rpms in flasks filled to one-fifth of volume at 30 °C.

Fermentations were also conducted in 0.5-L bioreactors (BIOSTAT Qplus system from Sartorius Stedim North America Inc., Bohemia, New York, USA) containing 250–300 mL of biomass hydrolysates or rich RMG medium as described previously [4, 10]. Fermentations were conducted at 30 °C with continuous stirring (150–300 rpm) and sparging (150 mL/min; 100% N_2_), and pH was controlled at 5.8–6.0. Samples were periodically removed from the bioreactors for an OD_600 nm_ measurement to monitor cell growth and for HPLC-RID analysis of the concentration of glucose, xylose, ethanol, and other end products. Process ethanol yields represent the ethanol produced based on the amount theoretically possible from complete conversion of the glucose and xylose in each hydrolysate (0.51 g ethanol/g sugar).

Cells for RNA isolation (10 mL) were also collected into 15-mL conical tubes containing 1.25-mL ice-cold unbuffered phenol in ethanol (5%, v/v) [45] and pelleted by centrifugation at 10,000*g*, 4 °C, for 3 min, then flash frozen in dry ice-ethanol bath, and stored at 80 °C.

### DNA isolation and NGS genome resequencing

For the isolation of total DNA for genomic and plasmid resequencing, all strains were grown in RMG to mid-log phase. Total DNA (containing both genomic DNA and plasmid DNA) was isolated using MasterPure DNA isolation Kit (Epicentre, Madison, Wisconsin, USA) or CTAB isolation protocol [46]. Samples were submitted to University of Wisconsin-Madison Biotechnology Center (http://www.biotech.wisc.edu/) for Illumina sequencing with paired-end 250 bp sequencing and Mate-Pair sequencing.

Alternatively, at NREL, genomic DNA was extracted from *Z. mobilis* cells grown in either RMG or RMX using Qiagen DNAEasy Kit and sent to the University of Utah for next-generation sequencing using Illumina Hiseq 2000 after samples passed the quality requirements. NGS-based genome resequencing using Illumina Hiseq 2500 was performed at Genewiz (HangZhou, China) for samples sent from Hubei University, China.

### Genome assembling

Nextera mate-pair reads were processed using NextClip v.1.3.1 [47], with the default parameters. The resulting A, B, and C type trimmed mate-pairs were kept. The short-pair reads were trimmed using Trimmomatic v 0.32 [48] and using the Illumina TruSeq adapters with parameters: seed mismatches of 2, palindrome clip threshold of 30, simple clip threshold of 10, headcrop of 0, and minlen of 10. Trimmed short-pair reads were normalized using insilico_read_normalization.pl provided by the Trinity software package [49] using KMER_SIZE = {21, 23, 24, 25, 27, 29} and max_cov = {10,15, 25, 35, 50}. For each setting of KMER_SIZE and max_cov, a genome assembly was generated using ALLPATHS-LG v.44837 (http://software.broadinstitute.org/allpaths-lg/blog/) [50, 51] using the trimmed/normalized short-pair reads and the NextClip processed mate-pair reads. Assembly statistics were collected using assemblathon_stats.pl [52]. Gaps in the scaffolds were then filled in by mapping the reads to the ALLPATHS-LG scaffolds and SeqMan NGen software (DNAstar Inc., Madison, Wisconsin, USA).

Alternatively, at NREL and Hubei University, the quality of FASTQ genome resequencing data was checked using FastQC program, data passing the quality control were imported into CLC Genomics Workbench for data analysis to identify the potential small genetic changes such as SNP and Indels. Briefly, sequencing reads after trimming to remove reads with poor quality were mapped against the reference genome sequence followed by resequencing analysis using both probabilistic variant detection and quality-based variant detection tools. The variants identified were filtered to remove those with less than 80% confident frequency level [53] before comparing among different strains. In addition, the fastq files were combined for each strain and used for de novo assembly using CLC Genomics Workbench. Contigs were compared with published genome sequence and primer sets were designed for primer walking to confirm the correct orientation and sequence of the plasmid and chromosomal sequences.

### Gene annotation

Blast2Go (https://www.blast2go.com) and InterProScan (https://www.ebi.ac.uk/interpro/search/sequence-search) were used to collect the latest comprehensive protein function information by submitting the translated sequences to InterPro-supported databases. Additional gaps in functional annotation were partially filled by searching SMART protein domain database (http://smart.embl-heidelberg.de) and using this information in conjunction with information on operon structure to gain insights on possible function of unannotated genes.

### Plasmid copy number measurement

Plasmid copy numbers were measured by quantitative PCR and comparison of plasmid-based amplicons to a chromosome amplicon at the replication terminus (*ter*), assuming the copy number of the chromosome replication terminus is one. Amplicons corresponding to randomly chosen segments of pZM32, pZM33, pZM36, and pZM39, the chromosome replication terminus, and, for reference, the chromosome replication origin (90–150 bp; Additional file 3: Table S2) were identified using Primer3 (http://primer3.ut.ee) [54]. The amplification fidelity and efficiency of each PCR amplicon were assessed prior to selecting the amplicons used for the assay. DNA segments containing the amplicons were cloned into pUC19 to create calibrator plasmids by Gibson assembly (Additional file 3: Table S2). Each calibrator plasmid contained two DNA segments, a DNA segment from one of the *Z. mobilis* plasmids with 250 bp flanking the PCR amplicon and an adjacent segment from the *Z. mobilis* chromosome replication terminus with 250 bp flanking the PCR amplicon. The sequences of the entire inserted segments were confirmed by Sanger sequencing using M13 forward and reverse primers.

For plasmid copy number experiments, *Z. mobilis* strains were grown overnight in RMG medium. Cells were then pelleted and diluted to apparent OD_600 nm_ value around 0.10–0.15 in RMG and grown either aerobically in a baffled flask at 30 °C and 250 rpm or anaerobically in a 50-mL tube or bottle with the cap open 1/2 of a turn at 30 °C. At harvest, 0.6–1.2 mL cells were pelleted, resuspended in 100–200 μL 100 mM Tris–HCl, pH 8.0, lysed at 95 °C for 20 min, and stored at − 20 °C. For each condition, replicate samples were then subjected to quantitative PCR using each amplicon to determine the plasmid copy numbers relative to chromosome replication terminus, with correction for amplification efficiency using the calibrator plasmids. qPCR was performed with a 7500 Real-Time PCR System (Applied Biosystems, Foster City, CA, USA) using SYBR Green JumpStart Taq ReadyMix (Sigma, St. Louis, MO, USA) in a 25-μL reaction volume using 1-μL 25 μM forward and reverse primer and 1.5-μL whole cell lysate. Cell lysates were diluted 1/10, 1/20, 1/40, 1/80, and 1/160 in the replicates.

Plasmid copy numbers (PCN) were determined by ∆∆Ct method [55], using the following formulas:

$${\text{Plasmid copy number }}\left( {\text{PCN}} \right)\, = \,2^{{ - \Delta \Delta {\text{Ct}}}}$$

$$\Delta \Delta {\text{Ct}}\, = \,\Delta {\text{Ct }}\left( {\text{sample}} \right) - \Delta {\text{Ct }}\left( {\text{calibrator plasmid}} \right);$$

$$\Delta {\text{Ct }}\left( {\text{sample}} \right)\, = \,{\text{Ct }}\left( {{\text{target }}1} \right) - {\text{Ct }}\left( {{\text{reference }}1} \right);$$

$$\Delta {\text{Ct }}\left( {\text{calibrator plasmid}} \right)\, = \,{\text{Ct }}\left( {{\text{target }}2} \right) - {\text{Ct }}\left( {{\text{reference }}2} \right);$$

$${\text{target }}1\, = \,{\text{target region from plasmid}};$$

$${\text{reference }}1\, = \,{\text{terminator region of}}\;Z. \, mobilis{\text{ chromosome}};$$

$${\text{target }}2\, = \,Z. \, mobilis\;{\text{plasmid target region on calibrator plasmid}};$$

$${\text{reference }}2\, = \,{\text{terminator region of}}\;Z. \, mobilis\;{\text{chromosome on calibrator plasmid }}\left( {\text{DH10B transformed with calibrator plasmid}} \right).$$

### RNA isolation and RNA-Seq data analysis

Cells for RNA isolation were collected using an ice-cold ethanol/phenol stop solution, and RNA was extracted using hot phenol method as described previously [45]. RNA samples were submitted to DOE-Joint Genome Institute (JGI: http://jgi.doe.gov/) for RNA-Seq. Ribo-Zero™ rRNA Removal Kits from Epicentre was used for ribosomal RNA (rRNA) depletion. Alternatively, at NREL, rRNA was removed by using a customized rRNA depletion kit developed together with Life Technologies using species-specific rDNA probes, and the rRNA-depleted total RNA was sent to University of Utah for RNA-Seq using illumina directional RNA-Seq library construction and sequencing. The rRNA-depleted total RNA was also used as the template for cDNA synthesis and qPCR following previous protocol [10, 56, 57] to determine the rRNA depletion efficiency.

Sequencing reads that passed standard Illumina quality control procedures were used for the alignment and counting. Transcriptome alignment was performed using Bowtie version 0.12.7 [58], followed by probabilistic counting with RSEM version 1.2.4 [59]. Strand-specificity of the libraries (generated with dUTP protocol) was accounted for as “–forward-prob 0” RSEM parameter. At NREL, the quality of RNA-Seq fastq data was checked using FastQC program (http://www.bioinformatics.babraham.ac.uk/projects/fastqc/); data passing the quality control were imported into CLC genomics workbench (https://www.qiagenbioinformatics.com/products/clc-genomics-workbench/), and the reads were trimmed for nucleotides with quality score less than 30 before RNA-Seq analysis to get the RPKM values of protein-coding genes and rRNA genes. The RPKM values were then imported into JMP genomics (https://www.jmp.com/en_us/home.html) and log_2_-transformed before statistical analysis similar to microarray-based transcriptomic analysis was performed [10, 56, 57].

For data normalization and testing for differential expression, Pearson correlation between vectors of counts that belong to biological replicates was employed to detect outlier libraries. All the retained libraries show the inter-replicate Pearson correlation of at least 0.95. Features representing rRNA and tRNA were removed from the matrix before the normalization. The matrix of counts for the entire set of libraries was pre-normalized as a pool using linear scaling to equilibrate the median values across the libraries. The pool of gene length- and library size-normalized values (FPKM) was additionally quantile-normalized via Bioconductor “affy” package [60]. The differential expression testing was performed using EBSeq [41], the Empirical Bayesian-based differential expression calling package. Genes with posterior probability of differential expression above 0.95 were subject to further filtering based on the quantile-normalized FPKM matrix, using a critical coefficient [61] cutoff of 1.5. Genes that pass both of the two selection criteria were called responsive and used in the functional analysis stage. For estimating replicon-wide relative expression, FPKM values for the chromosome and plasmids were generated using the sum of all non-rRNA, non-tRNA counts mapped to the respective replicon and the total length of the non-rRNA, non-tRNA features on the replicon.

The data structure was visualized using both hierarchical clustering and t-distributed stochastic neighbor embedding (t-SNE) [39]. For gene set enrichment analysis (GSEA), gene ontology (GO) associations were extracted from the results of InterProScan with translated plasmid ORF sequences. Cluster-based enrichment test with sets of responsive genes (FDR < 0.05) was performed with goseq package [62] from Bioconductor (http://bioconductor.org), the two gene-level statistics summarization tests—Fisher’s combined probability test [63] and summarization of median gene-level fold changes—using piano package [64] from Bioconductor. Statistical significance of the enrichment tests was estimated with 100,000 data permutations.

### Proteomic sample preparation, quantitative proteome measurement, and data analysis

The proteomic sample processing and data analysis in Additional file 9: Table S8 has been reported previously at ORNL [15, 16]. We here described the method for GLBRC proteomic data shown in Additional file 10: Table S9. Ten milliliters of *Z. mobilis* cells was collected at different growth conditions by centrifugation at 15,000*g* for 3 min, and quickly frozen in ethanol/dry ice bath and stored at − 80 °C until use. Cells were lysed by suspension in 6 M guanidine hydrochloride (GnHCl), followed by addition of MeOH to 90%. Samples were centrifuged at 15,000*g* for 5 min. Supernatant was discarded and pellets were allowed to dry for ~ 5 min. Pellets were resuspended in 200 µL 8 M urea, 100 mM Tris (pH 8.0), 10 mM TCEP, and 40 mM chloroacetamide, then diluted to 2 M urea in 50 mM Tris (pH 8). Trypsin was added at an estimated 50:1 (protein to enzyme) ratio, and samples were incubated overnight at ambient temperature. Each sample was desalted over a PS-DVB solid phase extraction cartridge and dried down. Peptide mass was assayed with the peptide colorimetric assay.

For each analysis, 2 µg of peptides was loaded onto a 75 µm i.d. 30-cm-long capillary with an imbedded electrospray emitter and packed with 1.7 µm C18 BEH stationary phase. The mobile phases used were A: 0.2% formic acid and B: 0.2% formic acid in 70% acetonitrile. Peptides were eluted with in increasing gradient of acetonitrile from 0 to 53% B over 75 min followed by a 5 min 100% B wash and a 10-min equilibration in 0% B [65]. Eluting peptides were analyzed with an Orbitrap Fusion Lumos. Survey scans were performed at *R* = 60,000 with wide isolation analysis of 300–1350 *mz*. Data-dependent top speed (2 s) MS/MS sampling of peptide precursors was enabled with dynamic exclusion set to 45 s on precursors with charge states 2–6. MS/MS sampling was performed with 1.6 Da quadrupole isolation, fragmentation by HCD with NCE of 30, analysis in the Orbitrap at *R* = 15,000 with max inject time of 22 ms, and AGC target set to 2 × 10^5^.

Raw files were analyzed using MaxQuant 1.5.8.3. Spectra were searched using the Andromeda search engine against a target decoy database generated in house. As described previously [66, 67], label-free quantitation and match between runs were toggled on and the number of peptides measurements for each protein was set to 1. Default parameters were used for all other analysis parameters. Peptides were grouped into subsumable protein groups and filtered to 1% FDR, based on target decoy approach.

## Additional files

**Additional file 1: Figure S1.** Completion of plasmid sequences by primer walking with a list of the primers used for each plasmid (**A**). PCR amplification of ZM4 chromosome region containing a 2.4-kb fragment near ZMO0133 locus that is absent in previously reported ZM4 genome sequence (**B**). A schematic is shown detailing the location of primers used to PCR, and PCR products on agarose gel are also shown. **Figure S2.** Customized rRNA depletion kit was developed with Life Technologies for *Z. mobilis* mRNA enrichment, and RNA-Seq result of the percentage of rRNA, tRNA, and mRNA in *Z. mobilis* total RNA was calculated (**A**). qRT-PCR measurement of rRNA content before and after rRNA depletion of total RNA using the customized kit (**B**). rRNA reduction is reported as the fold change in the target rRNA in total RNA relative to depleted RNA. Measurements were collected in WT (*Z. mobilis* strain 33C derived from *Z. mobilis* 8b) and MT (a mutant strain of 33C) grown in either rich media with 5% glucose (RMG) or rich media with 5% xylose (RMX) and collected in two biological replicates. Error is reported as standard deviation. Residual rRNA contamination and rRNA depletion efficiency of samples described in (**B**) was detected by RNA-Seq (**C**). Error is reported as standard deviation. An example of pairwise replicate correlation of RNA-Seq pseudo read counts (i.e. log_2_ transformed following addition of La Place constant of 1) for two biological replicates after rRNA depletion (**D**). **Figure S3.** Heatmap of RNA-Seq data from 6% and 9% ACSH, anaerobic (AN) and aerobic (AE) conditions. Coloring by condition (left color bar) corresponds to the one used for the Fig. 4. Blue, NREL, fermentor with biomass hydrolysates; black, NREL, flasks with rich RMG medium; light grey, GLBRC, 6% ACSH; Orange, GLBRC, 9% ACSH; light green, Univ. Athens, anaerobic; dark green, Univ. Athens (UA), anaerobic; dark green, UA, aerobic. Top index bar shows expression clusters (see Additional file 6: Table S5 for gene-cluster assignments). Right annotation bar shows generalized factor that is applicable to experimental designs across all the 3 research centers: “Early” and “Late” are growth stages.

**Additional file 2: Table S1.** Plasmid gene annotation and functional analysis.

**Additional file 3: Table S2.** Primers, amplicons, and calibrator plasmids used for plasmid copy number determination.

**Additional file 4: Table S3.** RNA-Seq metadata.

**Additional file 5: Table S4.** Plasmid gene RNA-Seq counts.

**Additional file 6: Table S5.** Comparison of RNA-Seq from 6% and 9% ACSH.

**Additional file 7: Table S6.** Comparison of RNA-Seq from anaerobic and aerobic conditions.

**Additional file 8: Table S7.** Proteomic data of ZM4 in ethanol shock and sodium acetate stress conditions.

**Additional file 9: Table S8.** Proteomic data of ZM4 in anaerobic and aerobic fermentation in rich and minimal media.

**Additional file 10: Table S9.** Assignments of plasmid genes to expression clusters identified by inter-center expression meta-analysis (see Additional file 1: Figure S3).

## Abbreviations

RMG: glucose-rich medium for *Zymomonas mobilis*
ACSH: hydrolysate produced using AFEX-pretreated corn stover
