# Supplementary material for: Complete genome sequence and the expression pattern of plasmids of the model ethanologen Zymomonas mobilis ZM4 and its xylose-utilizing derivatives 8b and 2032
Source: Biotechnol Biofuels. 2018 May 2;11:125. doi: 10.1186/s13068-018-1116-x (PMC5930841; doi:10.1186/s13068-018-1116-x)
Supplement: Supplementary file 1 — Additional file 1: Figure S1. Completion of plasmid sequences by primer walking with a list of the primers used for each plasmid (A). PCR amplification of ZM4 chromosome region containing a 2.4-kb fragment near ZMO0133 locus that is absent in previously reported ZM4 genome sequence (B). A schematic is shown detailing the location of primers used to PCR, and PCR products on agarose gel are also shown. Figure S2. Customized rRNA depletion kit was developed with Life Technologies for Z. mobilis mRNA enrichment, and RNA-Seq result of the percentage of rRNA, tRNA, and mRNA in Z. mobilis total RNA was calculated (A). qRT-PCR measurement of rRNA content before and after rRNA depletion of total RNA using the customized kit (B). rRNA reduction is reported as the fold change in the target rRNA in total RNA relative to depleted RNA. Measurements were collected in WT (Z. mobilis strain 33C derived from Z. mobilis 8b) and MT (a mutant strain of 33C) grown in either rich media with 5% glucose (RMG) or rich media with 5% xylose (RMX) and collected in two biological replicates. Error is reported as standard deviation. Residual rRNA contamination and rRNA depletion efficiency of samples described in (B) was detected by RNA-Seq (C). Error is reported as standard deviation. An example of pairwise replicate correlation of RNA-Seq pseudo read counts (i.e. log2 transformed following addition of La Place constant of 1) for two biological replicates after rRNA depletion (D). Figure S3. Heatmap of RNA-Seq data from 6% and 9% ACSH, anaerobic (AN) and aerobic (AE) conditions. Coloring by condition (left color bar) corresponds to the one used for the Fig. 4. Blue, NREL, fermentor with biomass hydrolysates; black, NREL, flasks with rich RMG medium; light grey, GLBRC, 6% ACSH; Orange, GLBRC, 9% ACSH; light green, Univ. Athens, anaerobic; dark green, Univ. Athens (UA), anaerobic; dark green, UA, aerobic. Top index bar shows expression clusters (see Additional file 6: Table S5 for gene-cluster ass [file 13068_2018_1116_MOESM1_ESM.pdf]

Gap closure of plasmid sequences by primer walking

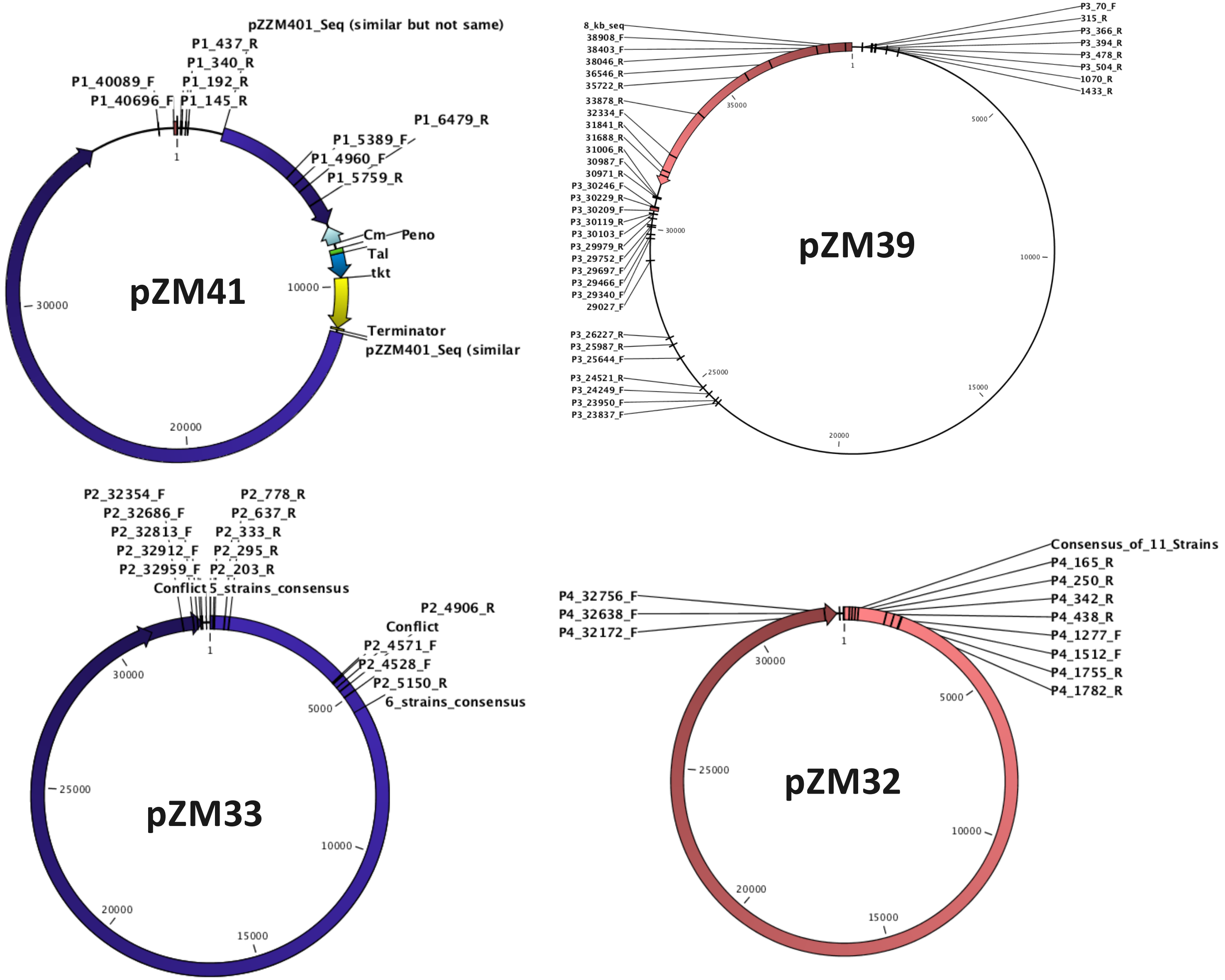

Fig. S1A

Identification of the missing chromosomal sequence using NGS

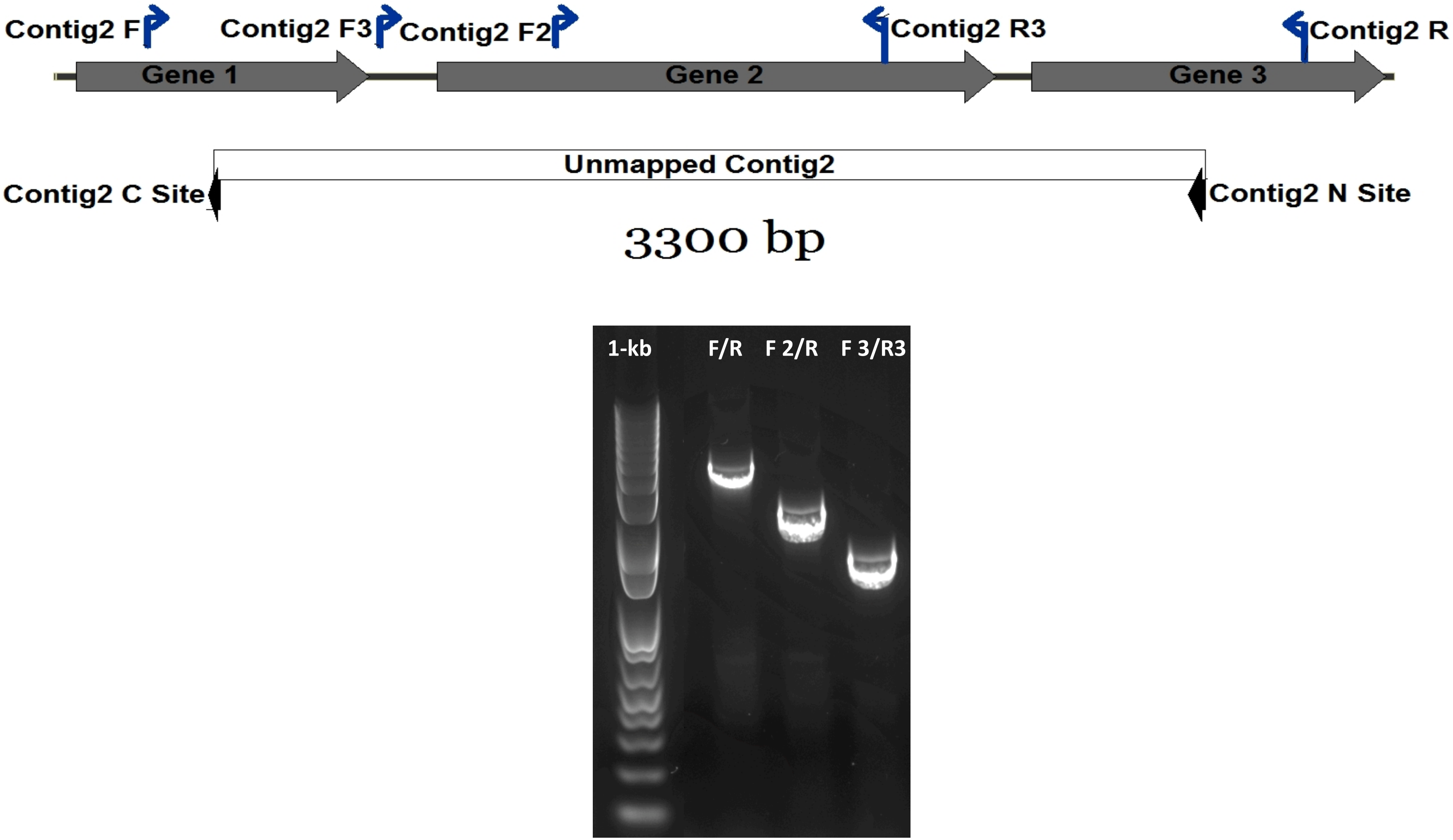

Fig. S1B

Efficient customized rRNA depletion kit developed for RNA-Seq

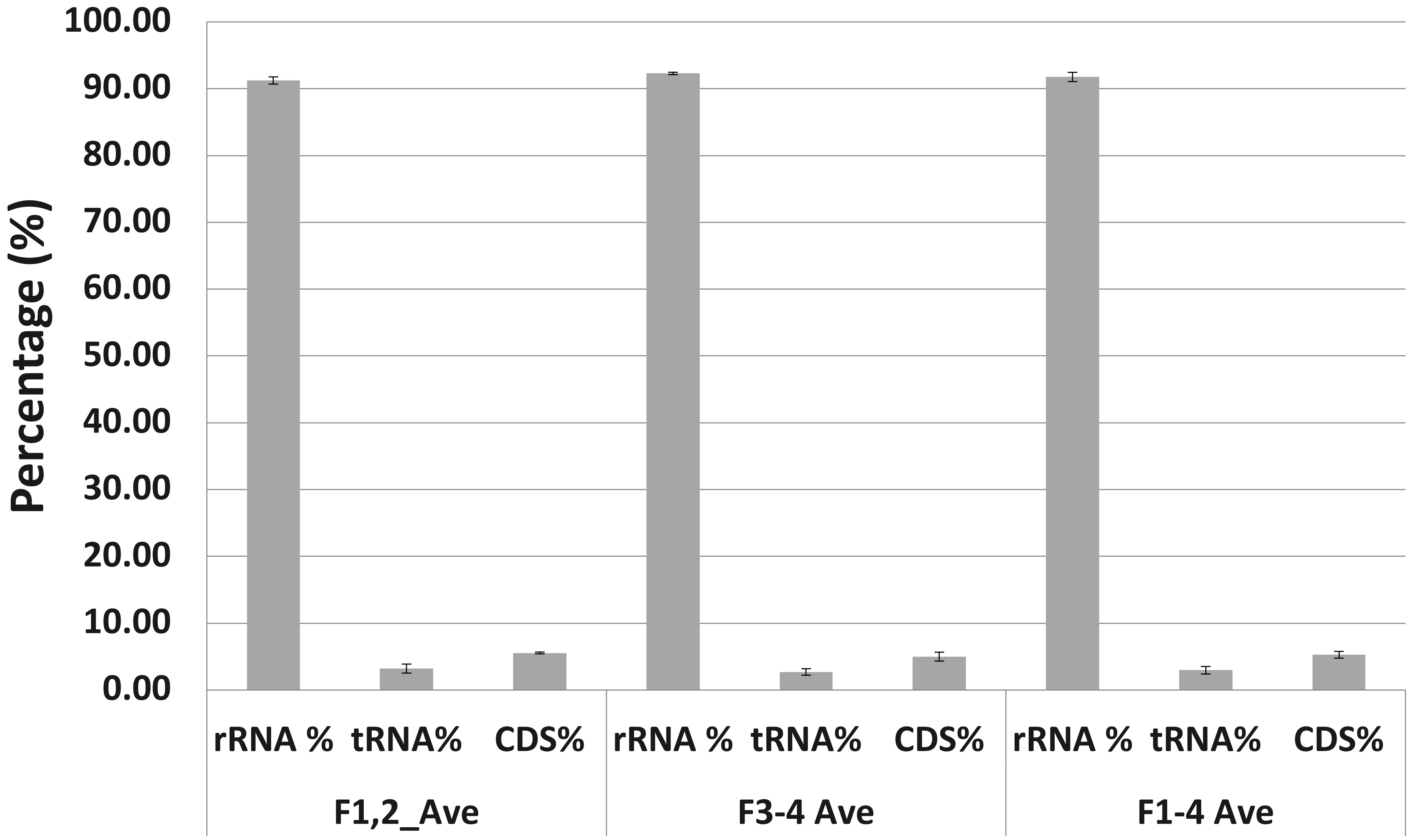

Fig. S2A

Confirmation of rRNA depletion efficiency by qRT-PCR

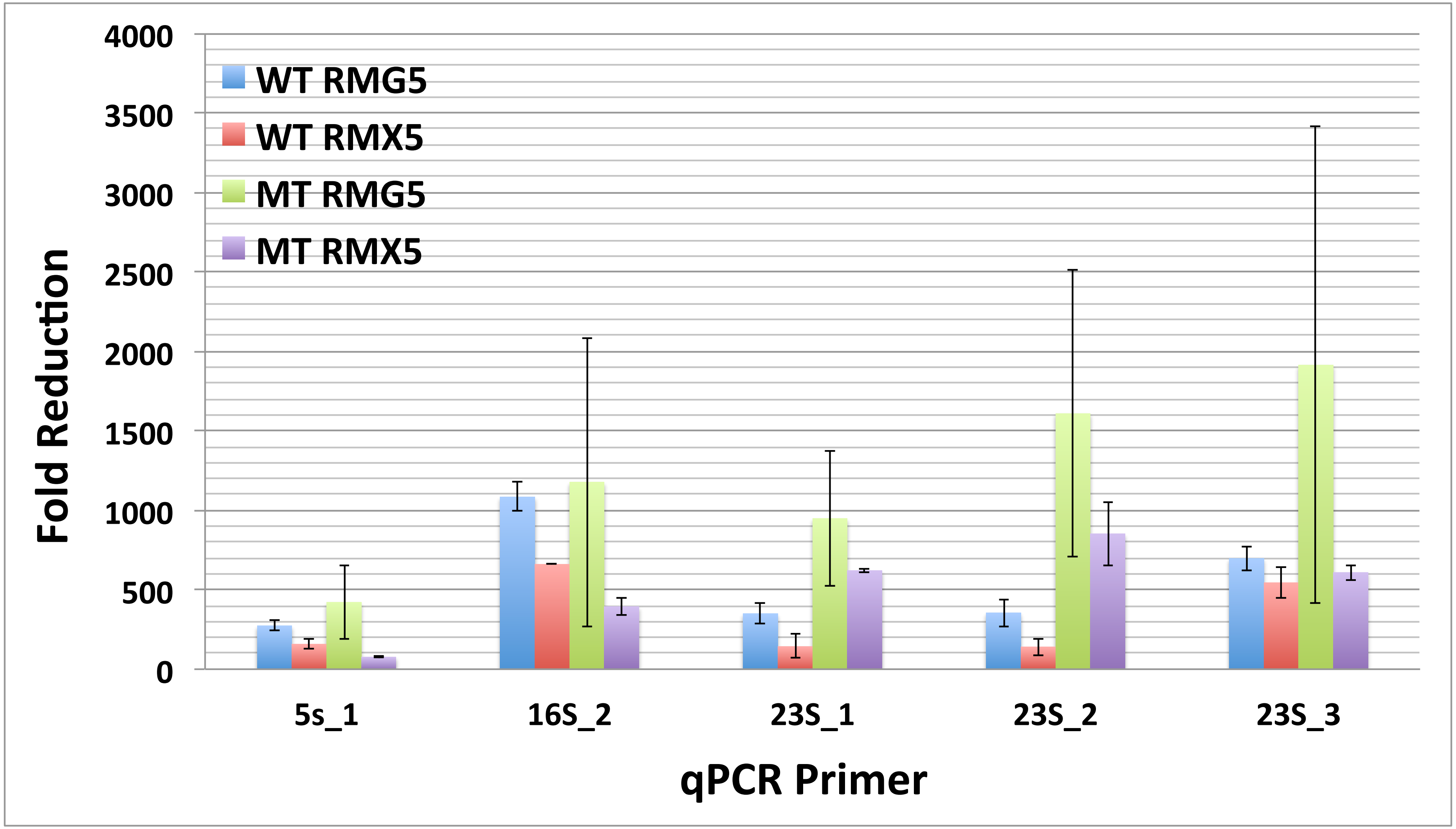

Fig. S2B

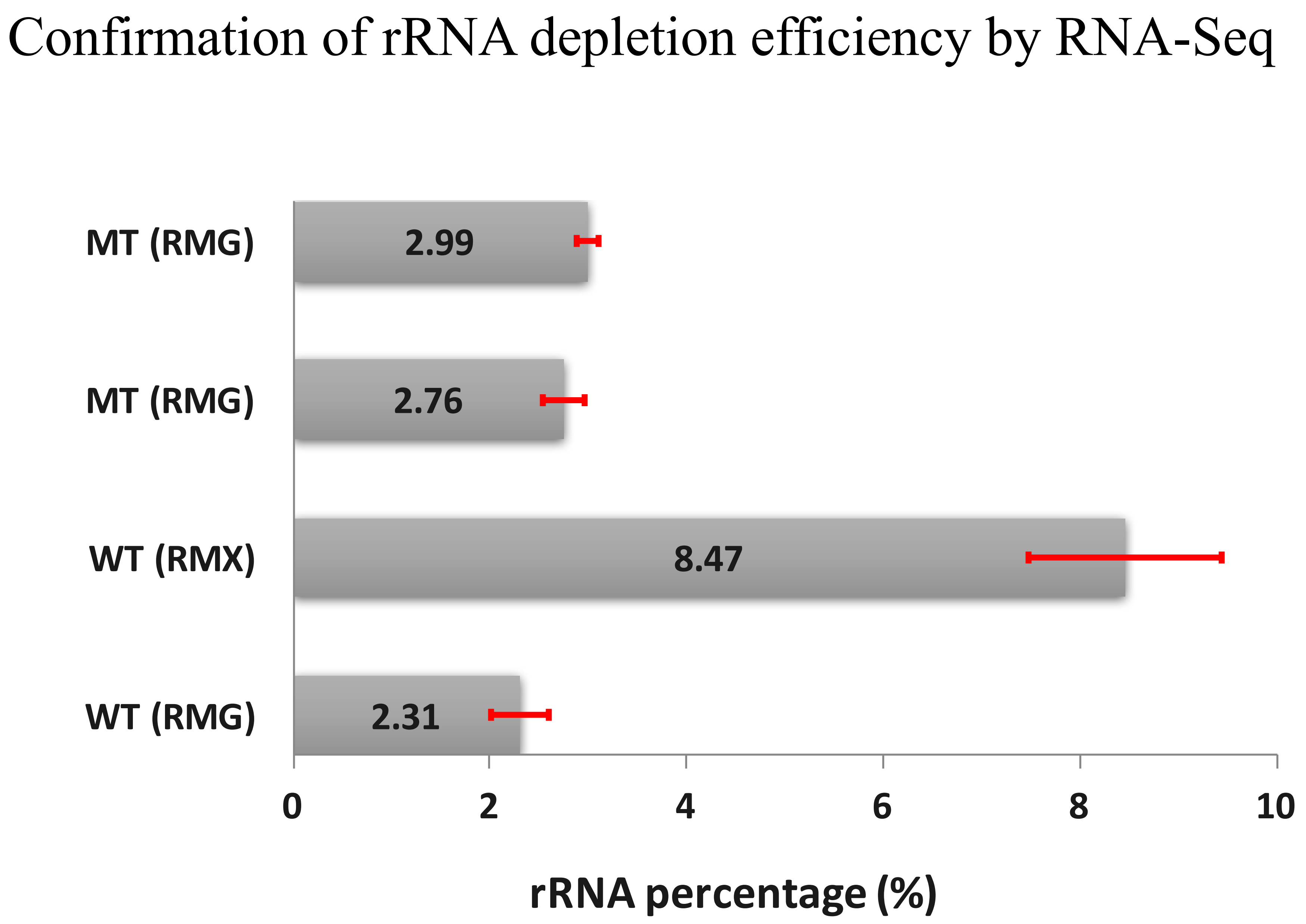

Confirmation of rRNA depletion efficiency by RNA-Seq
MT (RMG)
2.99
MT (RMG)
2.76
WT (RMX)
8.47
WT (RMG)
2.31
0
2
4
6
8
10
rRNA percentage (%)

Fig. S2C

Correlation between RNA-Seq replicates

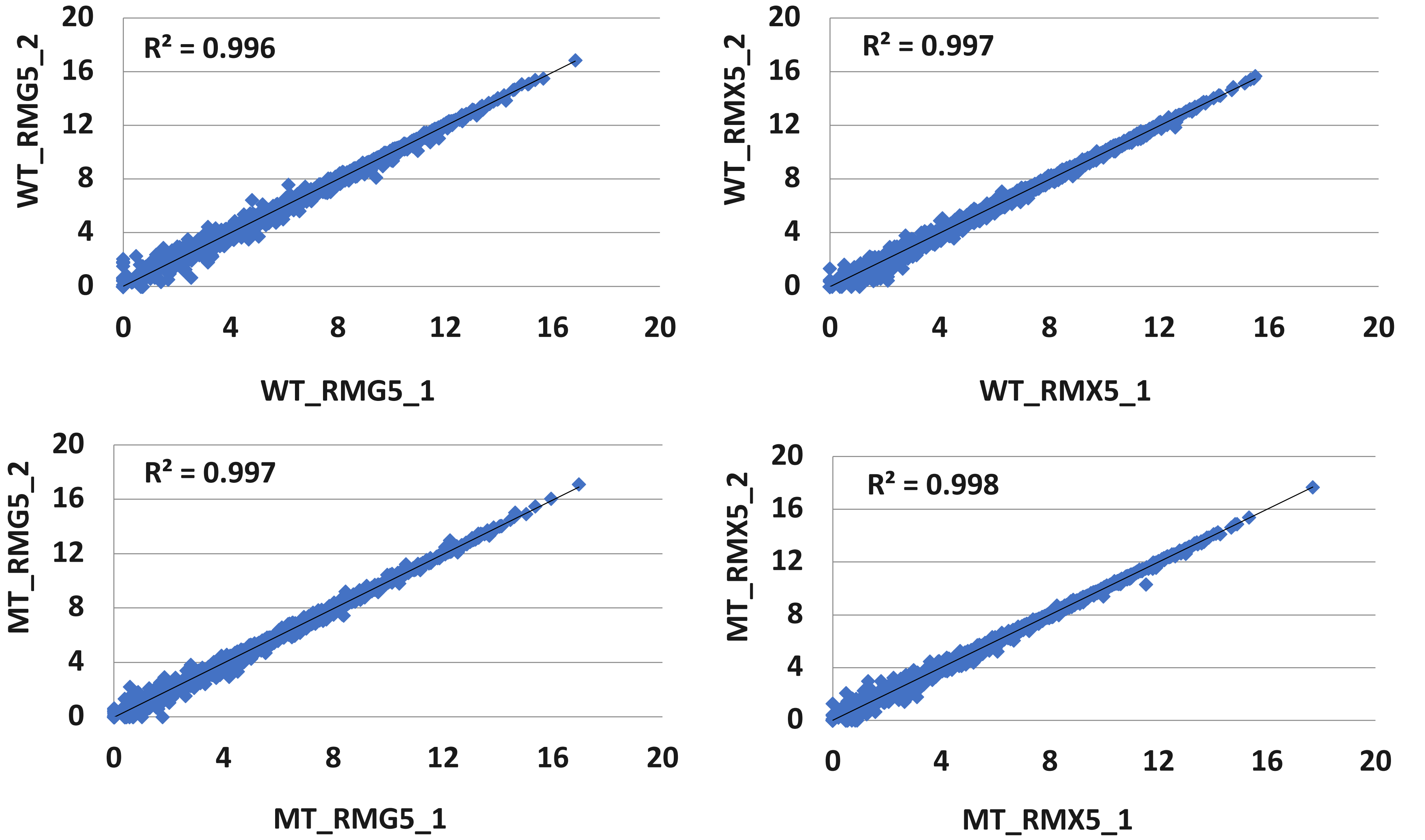

Fig. S2D

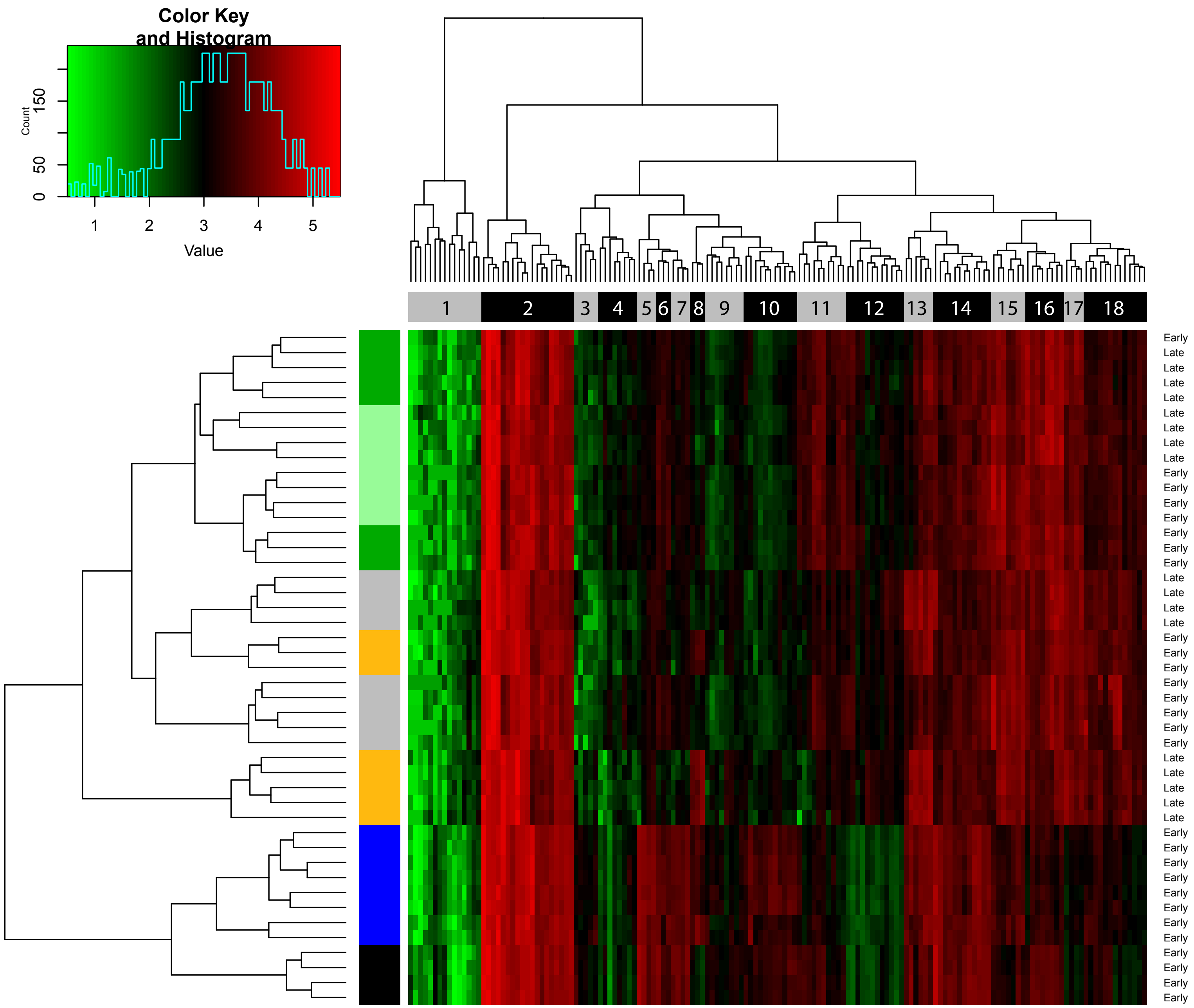

Fig. S3

Z. mobilis plasmid genes
