# Supplementary material for: Complete genome sequence and the expression pattern of plasmids of the model ethanologen Zymomonas mobilis ZM4 and its xylose-utilizing derivatives 8b and 2032
Source: Biotechnol Biofuels. 2018 May 2;11:125. doi: 10.1186/s13068-018-1116-x (PMC5930841; doi:10.1186/s13068-018-1116-x)
Supplement: Supplementary file 3 — Additional file 3: Table S2. Primers, amplicons, and calibrator plasmids used for plasmid copy number determination. [file 13068_2018_1116_MOESM3_ESM.docx]

**Table S2**. Primers, amplicons, and calibrator plasmids used for plasmid copy number determination.

| Primer# | Name | Sequence/Description | Target | Product |
| --- | --- | --- | --- | --- |
| *qPCR oligos* | |  |  |  |
| #10071 | pZM32_f | GGAATGATGCTGTGCTGTCC | pZM32_amp2b | 141 bp |
| #10072 | pZM32_r | CTTTAAGCTTTGTCTTTCTCACG | pZM32_amp2b | 141 bp |
| #10055 | pZM33_f | CAAAGTCCACCAGCGAGAC | pZM33_amp3 | 138 bp |
| #10056 | pZM33_r | ACGCTTCACCAGTTCTTCTG | pZM33_amp3 | 138 bp |
| #10057 | pZM36_f | CACGAGCTTTCTGATTGCTC | pZM36/41_amp1 | 91 bp |
| #10058 | pZM36_r | AAGGCTTGGCAATGACGAAG | pZM36/41_amp1 | 91 bp |
| #10059 | pZM39_f | CAATCGCAAAGCCTCGTTTC | pZM39_amp3 | 99 bp |
| #10060 | pZM39_r | CGGTGGGTGTCTCGAATAAC | pZM39_amp3 | 99 bp |
| #10063 | Zmter_f | ATTACAGGAGCAGAAAACGAATG | *Z. mobilis ter* (Zm4) | 157 bp |
| #10064 | Zmter_r | AACGGAATGGCGTGGATTTC | *Z. mobilis ter* (Zm4) | 157 bp |
| #10075 | Zmori_f | CGGCCAGACGAATGTGACC | *Z. mobilis ori* (Zm4) | 92 bp |
| #10076 | Zmori_r | AAATTGGTAGGTGCGGCAAC | *Z. mobilis ori* (Zm4) | 92 bp |
| *Gibson assembly oligos* | | |  |  |
| #10039 | 32gib_f | aaagaaccaCTGTCAGCTATAATGCTAAAG | pZM32_amp2b_gibson | pJG023* |
| #10040 | 32gib_r | ttgcatgcctgcaggGATTACTCAATGTCAGAATAGC | pZM32_amp2b_gibson | pJG023 |
| #10042 | 33gib_f | aaagaaccaAGAGGCCCCGAAAGCCTG | pZM33_amp3_gibson | pJG024 |
| #10043 | 33gib_r | ttgcatgcctgcaggTGTGCAGAACATGAGATTACGGC | pZM33_amp3_gibson | pJG024 |
| #10080 | 36gib_f | aaagaaccaGCCCAGAGCACGAGCTTT | pZM36/41_amp1_gibson | pJG025 |
| #10081 | 36gib_r | ttgcatgcctgcaggAGCAATGCGACTGATCAGATC | pZM36/41_amp1_gibson | pJG025 |
| #10106 | 39gib_f | aaagaaccaGTTCGGGTGTCATATAGG | pZM39_amp3_gibson | pJG026 |
| #10107 | 39gib_r | ttgcatgcctgcaggGATGCTTGTAATTATATAAAATACAATATTAC | pZM39_amp3_gibson | pJG026 |
| #10051 | origib_f | aaagaaccaGCGTATGAAAAATTTTATTAAAAAAGGAG | *Zmo ori*_gibson | pJG027 |
| #10052 | origib_r | ttgcatgcctgcaggGGCCAATTTTGAATCCCTTATTAAAAC | *Zmo ori*_gibson | pJG027 |
| #10037 | pUC19_f | agctcggtacccgggCAAGCAGAGTAAGACATTATAAAAAG | pUC19_gibson | pJG023-027 |
| #10038 | pUC32_r | gctgacagTGGTTCTTTGGGCTGAAATAG | pUC19_gibson | pJG023 |
| #10041 | pUC33_r | gggcctctTGGTTCTTTGGGCTGAAATAG | pUC19_gibson | pJG024 |
| #10079 | pUC36_r | ctctgggcTGGTTCTTTGGGCTGAAATAG | pUC19_gibson | pJG025 |
| #10105 | pUC39_r | acccgaacTGGTTCTTTGGGCTGAAATAG | pUC19_gibson | pJG026 |
| #10050 | pUCori_r | tcatacgcTGGTTCTTTGGGCTGAAATAG | pUC19_gibson | pJG027 |
| *Calibrator Plasmids* | | |  |  |
| #5023 | pJG023 | pUC19-derived calibrator plasmid with adjacent *ter* and p32 segments | | |
| #5024 | pJG024 | pUC19-derived calibrator plasmid with adjacent *ter* and p33 segments | | |
| #5025 | pJG025 | pUC19-derived calibrator plasmid with adjacent *ter* and p36/41 segments | | |
| #5026 | pJG026 | pUC19-derived calibrator plasmid with adjacent *ter* and p39 segments | | |
| #5027 | pJG027 | pUC19-derived calibrator plasmid with adjacent *ter* and *ori* segments | | |
